# Ensemble Deep Learning Denoising (EDLD) model and optimized OTSU segmentation for Alzheimer's disease diagnosis using MRI images

**DOI:** 10.3389/frai.2026.1743818

**Published:** 2026-05-25

**Authors:** S. Amuthan, N. C. Senthilkumar

**Affiliations:** School of Computer Science Engineering and Information Systems, Vellore Institute of Technology, Vellore, India

**Keywords:** Adaptive Denoising Autoencoder (ADAE), Alzheimer's disease, brain MRI images, Attention Guided Convolutional Neural Network (AGCNN), Deep Convolutional Neural Network (DCNN), deep learning, Gaussian Deep Belief Network (GDBN), stage prediction

## Abstract

Diagnosing Alzheimer's disease (AD) is necessary to determine treatment options. AD categorization using machine learning (ML) relies on difficult, manually specified features. The most important stage in AD diagnosis is denoising to restore image stability and quality. An ensemble image denoising technique that combines Attention Guided Convolutional Neural Network (AGCNN), Adaptive Denoising Autoencoder (ADAE), and Gaussian Deep Belief Network (GDBN) improves image denoising performance. The hybrid AGCNN reduces noise and aligns along the global route by combining global and local characteristics. In ADAE, the encoder learns picture representations using convolutional layers (CLs) while the decoder uses deconvolutional layers. In addition, the GDBN extends the standard Deep Belief Network (DBN) to Gaussian Restricted Boltzmann Machines (RBMs). Ensemble learning selects the approach with the greatest Peak Signal-to-Noise Ratio (PSNR) to integrate learning outcomes. After separating the background from the foreground by calculating the variances within the two groups, OTSU determines the threshold that minimizes the weighted sum of the variances. Levy Grasshopper Optimization Algorithm (LGOA) optimizes threshold selection by mimicking grasshopper swarming. VGG16, the DCNN model, is pre-trained for Alzheimer's datasets. The results are Sensitivity (SEN -95.86%), specificity (SPC - 94.93%), precision (PPV – 94.55%), F1-score (F1 – 95.21%), accuracy (ACC -95.87%), and Area Under the Receiver Operating Characteristic Curve (AUC – 96.45%) assess system and method performance.

## 1 Introduction

**Figure 1:**
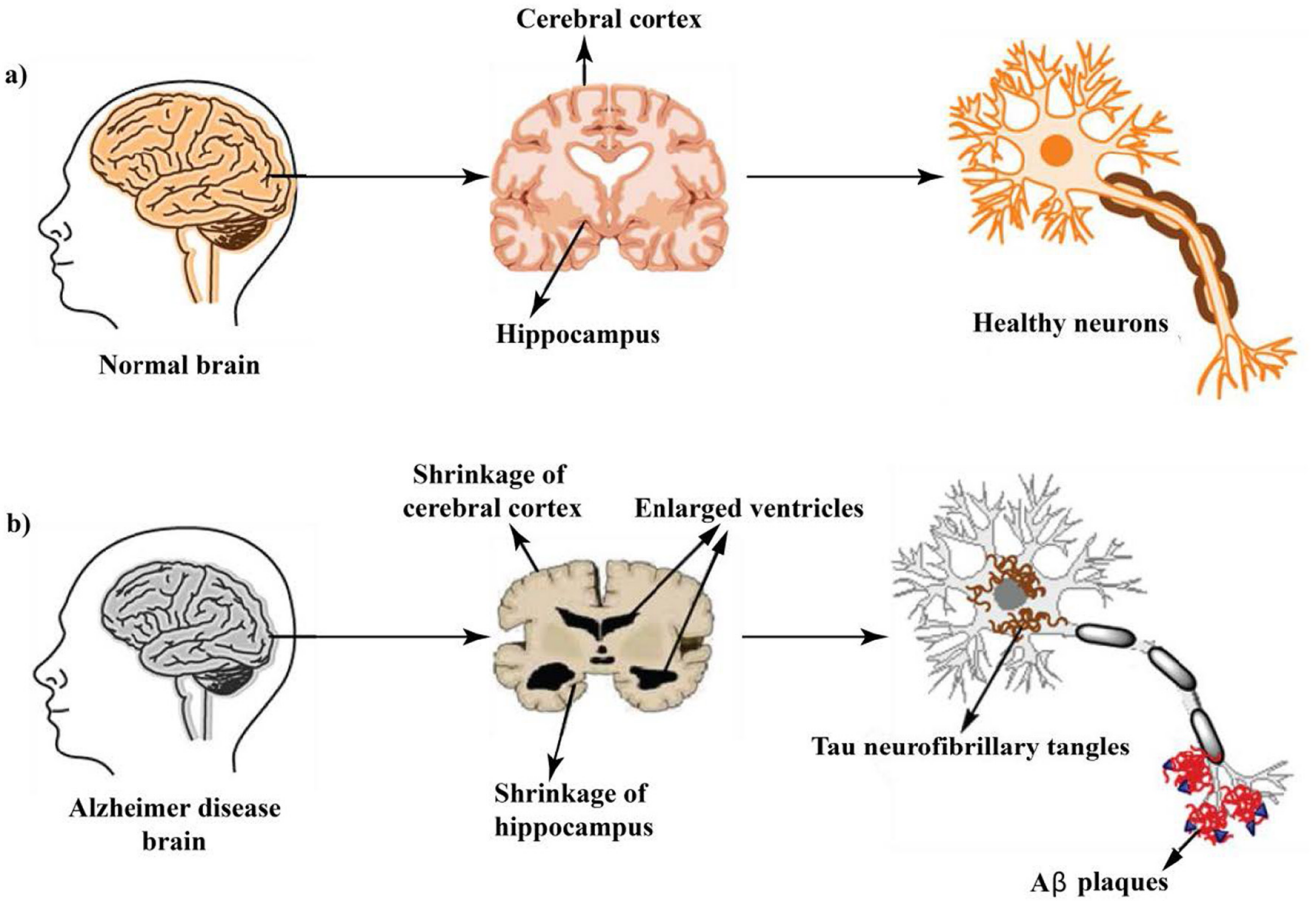
Physiological structure of the brain and neurons **(a)** healthy brain and **(b)** AD brain (Breijyeh and Karaman, 2020).

Alzheimer's disease (AD) is the most common neurodegenerative illness in the elderly population. Eventually, brain cells degrade and die, reducing brain volume and affecting numerous activities (Tzioras et al., 2023). Projections show that one in 85 people will have AD by 2050 (Kong et al., 2022). The most common is AD, a progressive neurological illness (Kong et al., 2022). Most often, neuritic plaques and neurofibrillary tangles in the neocortex and medial temporal areas indicate it. Figure 1 shows the formation of amyloid-beta (Aβ) peptides.

Traditional AD diagnosis has depended on cognitive and neuropsychological examinations after symptoms appear, with imaging and other diagnostic measures utilized as auxiliary measures (Breijyeh and Karaman, 2020). Unfortunately, most people with clinical symptoms are already in the moderate-to-severe stages of the illness, with no effective treatment (Malpetti and La Joie, 2022). AD pathophysiology involves structural changes in nerve cells. Depending on cognitive impairment, preclinical, moderate, and severe dementia is diagnosed clinically. Early on, long-term (LT) memory is generally intact, while short-term (ST) memory loss is sporadic.

Syphilis, vitamin B12 deficiency, cardiovascular disorders, and other causes of dementia are ruled out by imaging. As a result, early detection and treatment of Alzheimer's disease may improve patient outcomes and delay symptom development. AD is diagnosed using several methods, including: (1) clinical assessments that include a physical and medical history (Park et al., 2023); (2) Cognitive and neuropsychological tests (Park et al., 2023); (3) Neuroimaging techniques [magnetic resonance imaging (MRI) and computed tomography (CT)] to identify dementia causes like strokes, brain tumors, or brain degeneration (Bhujbal et al., 2023); (4) Biomarker analysis of tau and Aβ proteins (Bhujbal et al., 2023); (5) Genetic screening (Koriath et al., 2021); (6) Laboratory testing, including blood tests.

Medical imaging has been important for AD diagnosis and therapy (Weiskopf et al., 2021). Neuroimaging technologies, including functional magnetic resonance imaging (fMRI), positron emission tomography (PET), CT, and MRI, are essential for detecting brain abnormality. These imaging modalities offer consistent formats needed for research and clinical assessments. MRI scans may diagnose neurological illnesses due to their unique properties (Chaddad et al., 2018). MRI-derived characteristics such as GM and WM volumes, cortical thickness, and cerebrospinal fluid (CSF) levels assist in diagnosing and staging Mild Cognitive Impairment (MCI) and AD (Chaddad et al., 2018).

Segmenting brain MRI data over time tracks anatomical changes. Therefore, automated segmentation is crucial for precise, consistent findings. Computer-based MRI segmentation, visualization, and alignment on big datasets have helped medical professionals make more accurate judgments in recent years. AI, particularly machine learning (ML) and deep learning, is also crucial to AD research. MRI data is being analyzed using advanced AI algorithms to detect early, subtle AD symptoms, sometimes before they appear. These models may identify complex structural abnormalities in the brain that could indicate AD onset, enabling preclinical detection (Ding et al., 2019; Etminani et al., 2022). AI-based MRI analysis for monitoring illness progression is also advancing rapidly. Traditional ML systems can differentiate gray matter (GM), white matter (WM), and CSF. In individuals with Alzheimer's disease, abnormal brain tissues can also be identified through MRI segmentation.

However, obtaining the relevant imaging features for this process often demands complex feature engineering methods and domain-specific knowledge. OTSU's method is widely valued for its simplicity and speed (Merzban and Elbayoumi, 2019). With no prior knowledge of images, it automatically determines the optimal threshold by optimizing the division of an image's foreground and background regions. Recently, CNNs trained with prior knowledge have shown significant progress in automated identification of cognitive problems using brain MRI images. MRI-based analysis has successfully used existing deep learning models, such as AlexNet, VGG16, ResNet-18, ResNet-34, ResNet-50, SqueezeNet, and InceptionV3. Pre-processing improves the accuracy (ACC) and validity of brain scan data, thereby enhancing AD diagnostic accuracy. Image denoising, a major pre-processing step, aims to remove noise and reconstruct high-quality images to support imminent tasks, such as segmentation, disease diagnostics, detection, and classification. Deep learning has shown exceptional efficacy in image denoising due to its strong noise-reduction performance; however, overly deep networks in some methods can lead to increased computational demands (Ghose et al., 2020; Tiantian et al., 2024). Enhancing image quality during pre-processing significantly influences the effectiveness of AD diagnosis.

Novel contributions of this study are described as follows:

- Ensemble Deep Learning Denoising (EDLD): The proposed hybrid denoising framework integrates the Attention-Guided CNN (AGCNN), Adaptive Denoising Autoencoder (ADAE), and Gaussian Deep Belief Network (GDBN) to enhance MRI image quality robustly, outperforming single-model methods in image quality and visual fidelity.
- Optimized OTSU Segmentation: OTSU thresholding is combined with the Levy Grasshopper Optimization Algorithm (LGOA), which improves segmentation accuracy by adaptively determining optimal thresholds for foreground-background separation in MRI images.
- Efficient and Practical Framework: The proposed system is designed to achieve high classification performance using modest computational resources, demonstrating practical applicability in clinical or resource-limited environments.
- Evaluation with Statistical Validation: The study provides a thorough evaluation using sensitivity (SEN), specificity (SPC), precision (PPV), F1-score, accuracy (ACC), and AUC as statistical validation metrics, and includes comparisons with existing state-of-the-art (SOTA) methods.

## 2 Literature review

MRI-based image improvement models proposed by Kidoh et al. (2020) include the Denoising CNN (DnCNN), Shrinkage CNN (SCNN), and deep learning-based reconstruction. In particular, they used two-dimensional (2D) T2-weighted images, 2D fluid-attenuated inversion recovery (FLAIR), and three-dimensional (3D) Magnetization-Prepared Rapid Acquisition with Gradient Echo (MPRAGE) sequences collected from 15 healthy participants using the Number of Image Acquisitions 2 (NAQ2) and NAQ5, two different image collection settings. Both experimental and clinical studies were conducted, and statistical analyses were performed. In the clinical analysis, the system also measured inter-observer agreement to assess consistency. The Denoising Deep Learning Reconstruction (dDLR) method achieved better image quality than SCNN and DnCNN. Except for Peak Signal-to-Noise Ratio (PSNR) in the FLAIR sequence, dDLR-NAQ2 demonstrated much better Structural Similarity Index Measure (SSIM) and PSNR values throughout all three MRI sequences in clinical assessments when compared to NAQ2 (*p* < 0.05). For all qualitative assessment criteria except for artifacts, dDLR-NAQ2 matched or exceeded the quality of NAQ5 and outperformed NAQ2 (*p* < 0.05). Inter-observer agreement ranged from substantial to almost perfect. The comparison among the three denoising techniques was based on the Structural Similarity Index Measure (SSIM) and PSNR using brain MRI images.

Sanjay and Swarnalatha (2024) presented an AD detection hybrid deep CNN model incorporating denoising via a Multilayer Perceptron (MLP) and pooling layers. To identify important characteristics from imaging or molecular information relevant to AD, this model utilizes deep CNNs. MLP-based denoising improves input data and decreases noise. The pooling-layer-based feature representation helps identify local and global trends, improving AD prediction. A hybrid deep CNN model with MLP-based denoising and a future AD prediction technique is supplied via pooling methods. This complete procedure provides more accurate and dependable findings, aiding early diagnosis and treatment.

Progressive image enhancement methods were investigated by Alwakid et al. (2024) to improve AD diagnosis using brain MRI images. Local picture contrast and resolution enhanced by ESRGA and CLAHE. These MRI image preparation methods improve quality and performance. MobileNetV2 and DenseNet121, both feature extraction (FE) experts, were used for classification. This strategy improves AD diagnosis by using deep learning (DL) to classify brain tissue pictures. The model's MobileNetV2 and DenseNet121 accuracy rates were 92.34% and 89.38%, respectively, indicating good dependability on Kaggle's MRI dataset. Such results demonstrate the potential of deep learning and image enhancement to accurately and quickly diagnose AD.

Liu et al. (2021) classified ADs using depth-wise separable convolution (DSC) in a Deep Separable CNN (DSCNN) architecture. The computational cost and parameter count are far lower than those of typical CNNs. The model's low power consumption makes it ideal for embedded and mobile applications. Experimental findings show that the DSC technique can detect AD using Open Access Series of Imaging Studies (OASIS) MRI data. The framework's ACC is improved via transfer learning (TL). This approach employs pre-trained AlexNet and GoogLeNet, achieving average accuracies 91.40 and 93.02%, respectively.

Alhassan et al. (2022) introduced OTSU Enhanced Fuzzy Elephant Herding Optimization (OTSU-EFEHO), a Fuzzy Elephant Herding Optimization-based segmentation method. In this method, EFEHO determines the best OTSU segmentation threshold. We also advocate a Dual Attention Multi-Instance DL Network (DA-MIDL) to identify AD and MCI early on. This approach produces linkages quicker than the usual OTSU method while maintaining segmentation quality, according to experiments. A real-time high-speed picture partitioning method is presented in the study. The DA-MIDL model also showed superior categorization and generalization.

Jiong et al. (2019) developed a fully automated method to segment FLAIR and T1-weighted MRI White Matter Hyperintensities (WMH) using standard image processing techniques and deep neural networks. A unique skip-connection U-Net (SC-U-Net) is incorporated into pre-processing to perform skull stripping, reduce on-brain tissue, and improve segmentation. Two state-of-the-art (SOTA) models were presented for WM Hyperintensities (WMH) segmentation, with SC-U-Net showing better performance, reduced loss, and quicker convergence. Qualitative and quantitative evaluations validated SC-U-Net's excellence. The studies showed skull-stripping's value.

Chen et al. (2019) presented Iterative Linearly Constrained Minimum Variance (ILCMV), a classification algorithm that extends detection-focused ICEM. ILCMV classified normal brain tissues and White Matter Hyperintensities using spatial filters. Multi-Class Iterative Constrained Energy Minimization (MCICEM) was also created for comparison. Synthetic images from the BrainWeb dataset are used for quantitative evaluation of the ILCMV method, while actual brain MRI scans are used for visual analysis. From a computational efficiency perspective, the Gaussian filter is most compatible with both ILCMV and MCICEM. ILCMV or MCICEM, in addition to a Gabor filter, provides the optimal classification accuracy, provided processing time is not a constraint. Furthermore, in artificial magnetic resonance images with noise levels significantly greater than previously observed, CSF, GM, and WM's average Dice Similarity Index (DSI) scores and volume measurements were 0.936, 0.948, and 0.975, respectively, when combined with Gaussian filters. Compared with WMH-detection-only techniques, ILCMV can simultaneously categorize both normal brain tissue and WMH lesions in MRI images. These results suggest that the approach has a high probability of accurately classifying tissues and lesions in MRI applications.

Li et al. (2019) obtain tissue segmentation by using membership functions that indicate various tissue types, derived by minimizing an energy function in the fuzzy C-means (FCM) framework. These membership functions are first subjected to a temporal consistency restriction in a vibrational model. This constraint is further converted into a vector-valued function, enabling the explicit calculation of the membership functions via a mathematically defined mapping. Inhomogeneities and temporal changes in image intensity are well captured by these vector-valued functions, which provide details about each tissue type's average intensities and inclined area. In the Baltimore Longitudinal Study of Aging (BLSA), the technique's performance is assessed using a benchmark dataset.

The DA-MIDL, developed by Zhu et al. (2021), can detect AD and MCI. The DA-MIDL design comprises three primary modules: Patch-Nets and spatial attention processes provide relevant characteristics for each structural MRI (sMRI) patch and indicate aberrant microstructural alterations in the brain. An attention-based Multi-Instance Learning (MIL) pooling layer adjusts each patch's contribution to provide a globally weighted representation of the full brain structure, and a global classifier with attention mechanisms analyzes the combined data to make precise AD classification decisions. Baseline structural MRI scans from 1,689 individuals from two separate datasets, the Australian Imaging, Biomarkers and Lifestyle (AIBL) project and the AD Neuroimaging Initiative (ADNI) DA-MIDL model evaluation. According to experimental results, the DA-MIDL framework effectively identifies key pathological regions and delivers superior classification accuracy and generalization capability compared to several modern techniques.

Sathiyamoorthi et al. (2021) proposed a CAD system for AD prediction using multiple algorithms. A 2D Adaptive Bilateral Filter (2D-ABF) is used for image restoration, as thermal effects in the scanning hardware often introduce significant noise in MRI images. To enhance image clarity and increase contrast and brightness, use the Adaptive Histogram Adjustment (AHA) technique. The adaptive mean-shift modified expectation-maximization (AMS-MEM) segmentation method highlights AD-affected brain areas. Multiple features are extracted using the 2D-GLCM. DL is used to categorize and detect disease-related picture phases based on selected parameters. Categorization using DCNN aids diagnosis. Experimental findings show that the suggested technique is more efficient and reliable than current methods.

Murugan et al. (2021) developed a CNN-based framework for detecting discrete AD features in MRI images. The model's ability to classify the ADNI dataset was used to assess ADs. Also, the MRI data available on Kaggle turned out to have a serious class imbalance problem. The proposed plan applies a comprehensive diagnostic approach across all four stages of dementia, yielding high-resolution probability maps of disease presence for localized brain structures. A Multilayer Perceptron is then applied to these maps to provide accurate, visually interpretable representations of the individual AD risk. To address class imbalance, the dataset is balanced to ensure equal distribution of samples across all classes. They introduce a new architecture, DEMentiaNETwork (DEMNET), to detect different stages of dementia based on MRI data.

Asaduzzaman et al. (2025) used an MRI dataset to train the Alzheimer Recognition Ensemble Network (ALZENET) to discriminate among the various AD phases. The collection contains 6,400 MRI images, divided into four groups. Oversampling was implemented with SMOTE to reduce the large class imbalance. A multilayered CNN ensemble model employing VGG16, InceptionV3, and ResNet50 beat earlier AD classifiers. Additionally, a deep learning-powered internet software was created to assist doctors in identifying AD phases. Early and correct diagnosis may assist patients using this method.

Fathi et al. (2024) offer dataset gathering, pre-processing, model construction, ADNI data testing, and local dataset testing. The committee decided to compare ensemble configurations after a debate. The ensemble model used the six top CNN-based classifiers. The ensemble technique performed well on the local dataset, with a three-class classification accuracy of 88.46%.

Gharaibeh et al. (2023) propose a novel method for Alzheimer's disease detection using MRI images. Image noise is eliminated with the Hybrid Kuan Filter and Improved Frost Filter (HKIF) method during pre-processing. The Geodesic Active Contour (GAC) approach effectively eliminates non-brain tissues during skull stripping, boosting detection precision. This approach improves the non-uniformity in bias field correction intensity using Expectation-Maximization (EM). The Swin Transformer-based Segmentation with Modified U-Net and Generative Adversarial Network (ST-MUNet) segments after pre-processing. Using cortical thickness, color, and texture, and restricting features to distinguish GM, WM, and CSF from brain pictures, enhances segmentation accuracy.

## 3 Proposed methodology

**Figure 2:**
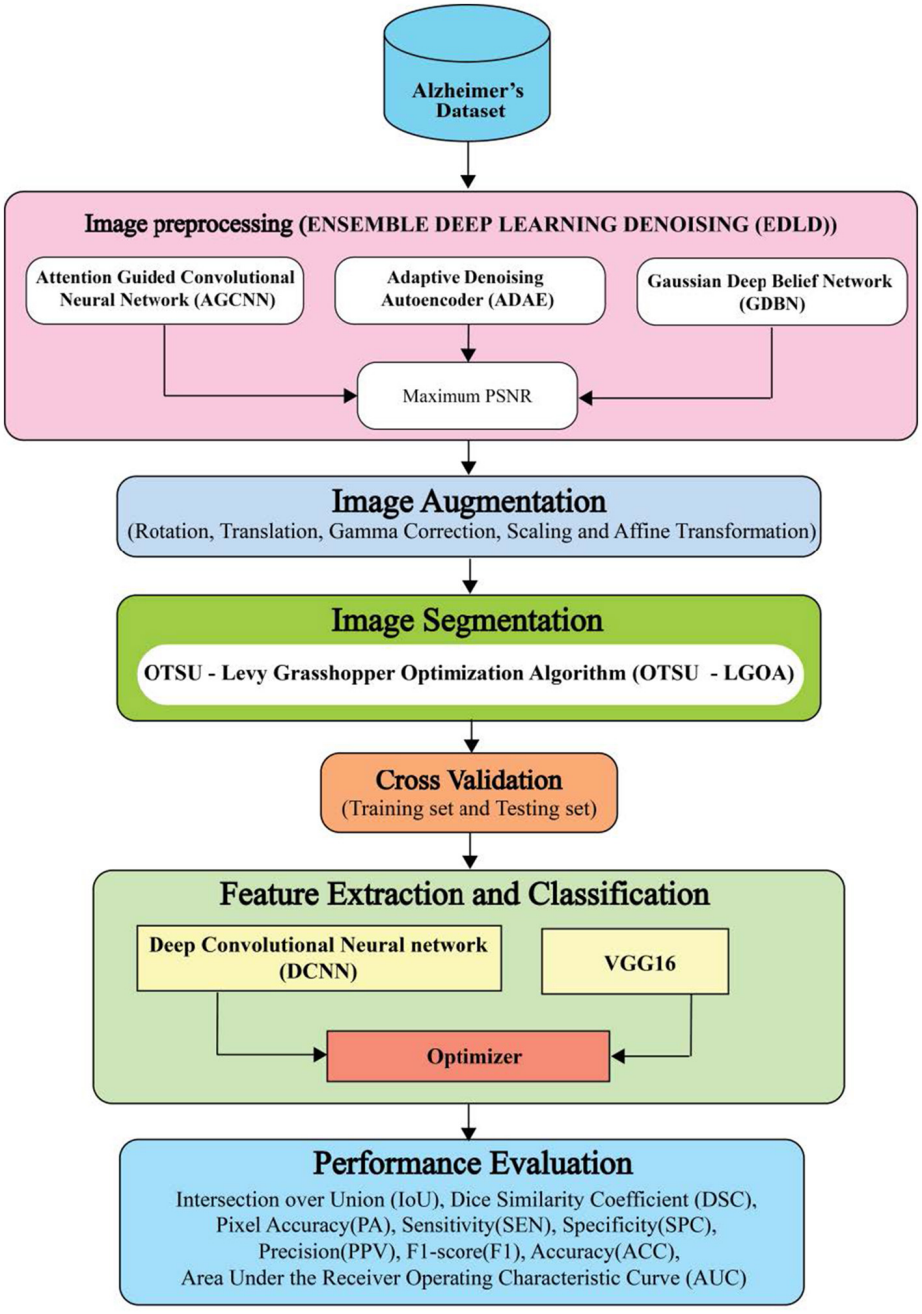
Overall process of the proposed system.

An ensemble image denoising strategy uses the Attention-Guided Convolutional Neural Network (AGCNN), Adaptive Denoising Autoencoder (ADAE), and Gaussian Deep Belief Network (GDBN) to reduce noise and image denoising. The OTSU method determines the best background-foreground threshold. It calculates the variance within each of the two clusters. It uses the Levy Grasshopper Optimization Algorithm (LGOA) to identify the threshold value that minimizes the weighted sum of these variances. DCNN model, the Visual Geometry Group (VGG16) is the pre-trained model for the Alzheimer's datasets. Figure 2 shows the overall framework of the proposed system.

### 3.1 Dataset collection

OASIS MRI dataset (https://sites.wustl.edu/oasisbrains/), which consists of 86,437 brain MRI images. The images have been divided into four classes based on Alzheimer's progression. The dataset aims to provide a valuable resource for analyzing and detecting early signs of Alzheimer's disease. Table 1 classifies dementia as Moderate Demented (MOD), Very Mild Demented (VMD), Non-Demented (NOD), and Mild Demented (MD). The resulting dataset size is 1.3 GB.

**Table 1:** Dataset details.

Classes	Training images	Testing images	Total
MD	4,726	276	5,002
MOD	420	68	488
NOD	65,563	1,659	67,222
VMD	12,567	1,158	13,725
Total	83,276	3,161	86,437

### 3.2 Image pre-processing and denoising

The main image pre-processing steps are transformation and normalization. During the transformation, all photos are reduced to 64 × 64 pixels. Training performance depends on downsizing the picture, which speeds up model learning. After resizing, photos are normalized separately. This map converts pixel values from 0 to 1 to 0 to 255. Transformed and normalized photos are stored in separate files for later use. Recently, DL-based image denoising techniques have performed well on test datasets with distributions as similar to those of the training data. Pre-processing procedures have greatly improved these real-world denoising models. Recent years have seen significant improvements in data-driven picture denoising algorithms.

#### 3.2.1 Attention Guided Convolutional Neural Network (AGCNN)

**Figure 3:**
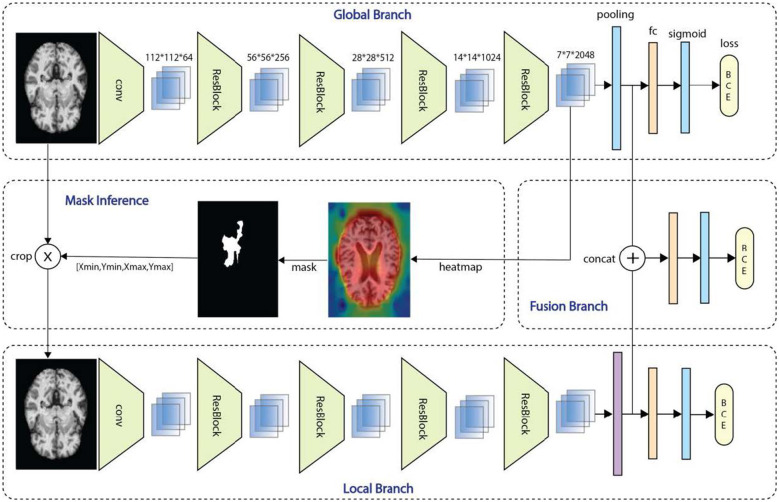
Attention Guided Convolutional Neural Networks (AGCNN) overall structure.

For picture denoising, an AGCNN is suggested. After concentrating on Alzheimer's disease-specific areas to minimize noise and improve feature alignment, AGCNN uses a global branch (GB) to recover discriminative information that the local branch (LB) missed. Initial training uses complete pictures for a global CNN branch. This global branch's attention heatmap is used to create a mask to isolate a crucial picture area. A distinct local CNN branch is trained in this constrained position. Combining the last pooling layers from the local and global branches refines the fusion branch. AGCNN enhances detection accuracy by better aligning Alzheimer's images and minimizing noise interference. As illustrated in Figure 3, the AGCNN architecture comprises three main branches: global, local, and fusion. All local branches (LBs) and global branches (GBs) serve as networks for categorization, designed to detect noise in an image. A CNN classifier trained on the entire image is first used to fine-tune the global branch for a particular Alzheimer's image. Next, a focused region is extracted from the global image based on attention, and this region is used to train the local branch for noise detection. In the final step, the output from the last pooling layers of both branches is merged to fine-tune the fusion branch.

Global and local branches: The global branch extracts key Alzheimer's-related features from the entire input image. The main model used in this branch is a modified version of ResNet-50. There are five downsampling blocks in all, a fully connected (FC) layer with 15 output dimensions for categorization, and a global max pooling layer. The FC layer's output vector, finally, is normalized by applying a sigmoid activation layer *p*_*g*_(*c*|*I*), as defined in Equation 1,


$$\begin{array}{l} {\tilde{\text{p}}}_{\text{g}}\left(\text{c}|\text{I}\right)=\frac{1}{\left(1+\exp\left(-\text{p}\left(\text{c}|\text{I}\right)\right)\right)} \end{array}$$ (1)

Here, I represent the world's image. The probability that I belong to the cth class, c ϵ {1,2,…,C}, is represented by the equation ${\tilde{p}}_{g}\left(c|I\right)$. Minimize the loss due to binary cross-entropy (BCE) in addition to maximizing the global branch's *W*_*g*_.


$$\begin{array}{l} L\left({\text{W}}_{\text{g}}\right)=-\frac{1}{\text{C}}\overset{\text{C}}{\underset{\text{c}=1}{\sum }}{\text{l}}_{\text{c}}\log\left({\tilde{\text{p}}}_{\text{g}}\left(\text{c}|\text{I}\right)\right)+\left(1-{\text{l}}_{\text{c}}\right)\log\left(1-{\tilde{\text{p}}}_{\text{g}}\left(\text{c}|\text{I}\right)\right) \end{array}$$ (2)

Here, C is the number of images, and *l*_*c*_ is the cth class's ground-truth label. Nevertheless, by focusing on the noisy region, it is anticipated that the LB would mitigate the disadvantages of relying solely on global perception. In particular, the convolutional network topology of the GB and the local branch is identical. It should be noted that while these two branches serve different objectives, they do not share weights. Show ${\tilde{p}}_{l}\left(c|I_{c}\right)$ as the local branch's probability score and *W*_*l*_ as its parameters. In this case, *I*_*c*_ is the LB's input image. Follow the global branch's normalization and optimization procedures. The fusion branch initially combines the GB and LB's Pool5 outputs. The final categorization is made possible by joining a 15-dimensional FC layer from the concatenated layer. It have${\tilde{\;p}}_{f}\left(c|\left[I,\;I_{c}\right]\right)$ as the probability score. Optimize *W*_*f*_ using Equation 2 and denote *W*_*f*_ as the fusion branch's parameters.

Attention-Guided Mask Inference: This study identifies discriminative noise regions across the entire image for detection using a binary mask. One approach to consider it as an attention process is to apply thresholding to the feature maps. The last convolutional layer's (CL's) kth channel output in ResNet-50 is *k* = 2,048, the spatial position (x, y) is stimulated, and is represented as $f_{g}^{k}\left(x,y\right)$ given a global image. The global branch is indicated by g. At position (x, y), first determine the activation values' absolute value $f_{g}^{k}\left(x,y\right)$. Then, by determining the greatest values along channels, the attention heat map *H*_*g*_ is produced in Equation 3.


$$\begin{array}{l} {\text{H}}_{\text{g}}\left(\text{x},\text{y}\right)=\underset{\text{k}}{\max}\left(\left|{\text{f}}_{\text{g}}^{\text{k}}\left(\text{x},\text{y}\right)\right|\right),\text{k}\in \left\{1,\ldots ,\text{K}\right\} \end{array}$$ (3)

The values directly show the significance of the activations for categorization in *H*_*g*_. To identify regions with high activation levels, create a binary mask M. If a spatial point (x, y) in the heat map exceeds τ, set M(x, y) = 1; see Equation 4:


$$\begin{array}{l} \text{M}\left(\text{x},\text{y}\right)=\{\begin{array}{cc} 1, & {\text{H}}_{\text{g}}\left(\text{x},\text{y}\right)>\tau \\ 0, & \text{else} \end{array} \end{array}$$ (4)

where the threshold τ determines the size of the attended region. A greater τ causes a smaller area, and vice versa. The horizontal and vertical axes' minimum and maximum coordinates [*x*_min_, *y*_min_, *x*_max_, *y*_max_] represent the region with the most connectivity. Image I is cropped in the input, and the local discriminative region *I*_*c*_ is enlarged to match I in size.

Training Strategy of AGCNN: The present study introduces a three-stage AGCNN training strategy.

Stage I. Modify ImageNet's pre-trained global branch network using global images.

Stage II. Mask inference with threshold τ produces the local image *I*_*c*_, which is then fine-tuned using the LB. Equation 1 normalizes ${\tilde{p}}_{l}\left(c|I_{c}\right)$ additionally. The weights of the GB remain the same when the LB is optimized.

Stage III. Let us use the Pool_5 layer for the global and local branches' outputs, which Pool denotes *l*_*g*_ and *Pool*_1_, respectively. For the last fine-tuning (FT) step, concatenate them and use Equation 1 to normalize the probability score ${\tilde{p}}_{f}\left(c|\left[I,\;I_{c}\right]\right)$. When improving the weights of the fusion branch, fixed weights pertain to the previous two branches. Through image alignment, rectification, and noise reduction, AGCNN improves noise performance.

#### 3.2.2 Adaptive denoising autoencoder (ADAE)

In ADAE, the encoder uses convolutional layers (CL) to learn pixel representations, while the decoder uses deconvolution layers to recover the data. Denoising is implemented using Adaptive Shrinkage Units (ASUs) in both the encoder and the decoder. To improve overall detection accuracy and to avoid overfitting, the network is denoised using dropout regularization. Neural networks that replicate input images are called autoencoders (Lore et al., 2017). In lower-dimensional implicit space, autoencoders are designed to decode an input image back to its original form. They are composed of an output, a hidden, and an input layer. The hidden layer separates the autoencoder's input and output layers, and acts as a bottleneck, since it recognizes that the input image is compressed and that the network's capacity is being used to learn it. Using Equation 5, this component applies non-linear transformations to convert a latent representation h from the input image x.


$$\begin{array}{l} h=f\left(x\right)=\varphi \left(Wx+b\right) \end{array}$$ (5)

The original image x is then restored by the decoder network using the latent representation h. An additional set of non-linear transformations is used in this procedure, with the decoder's bias vector, weight matrix, and activation function denoted by σ, W′, and b′, respectively, as expressed in Equation 6.


$$\begin{array}{l} \tilde{x}=g\left(h\right)=\sigma \left(W^{\prime }h+b^{\prime }\right) \end{array}$$ (6)

**Figure 4:**
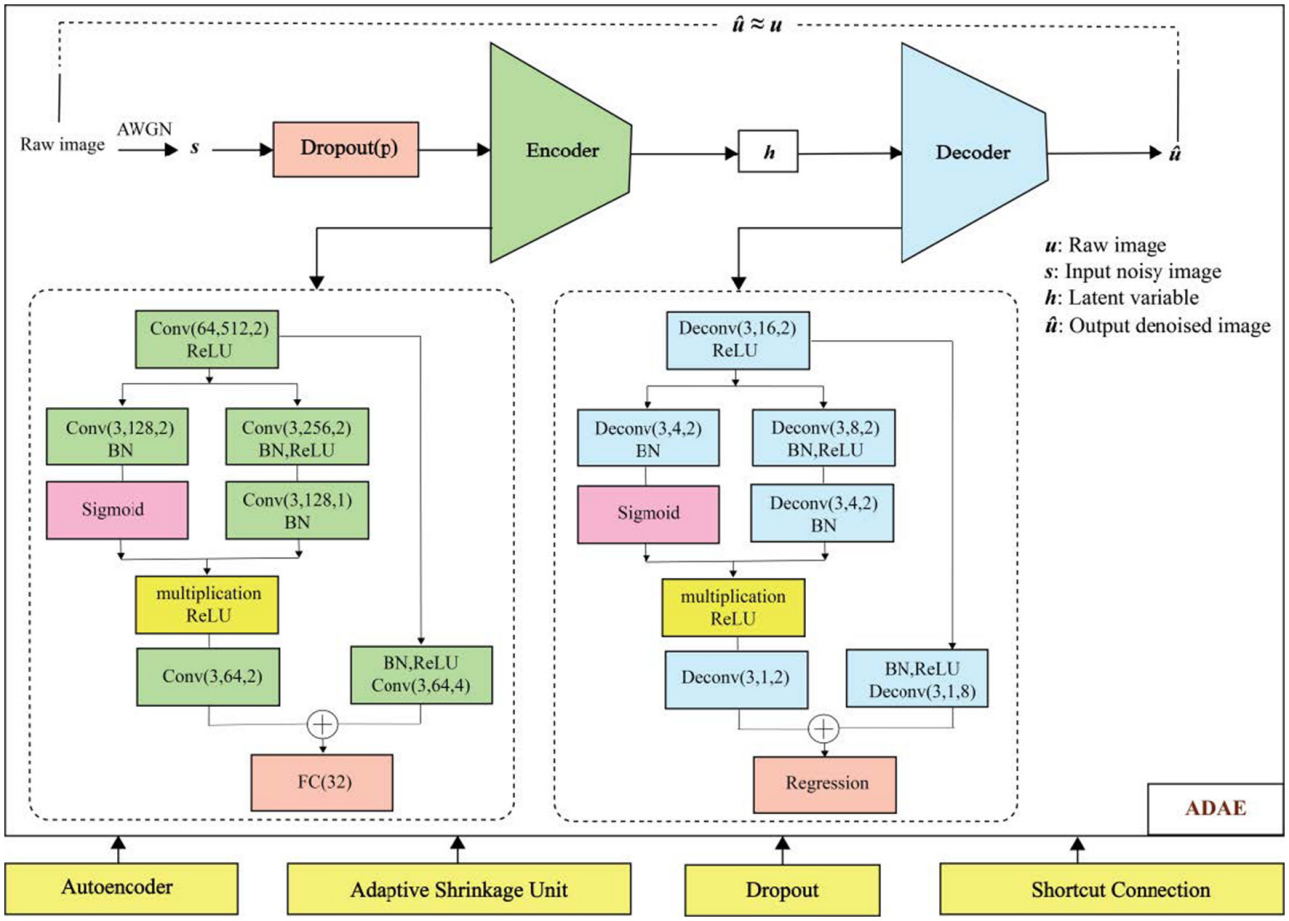
Overview of the proposed ADAE structure.

The ability of an autoencoder to accurately recreate the input is used to assess its performance. The distinction between the reconstruction x and the input x is measured by the reconstruction error (Lore et al., 2017), which is represented by L (x, $\tilde{x}$). The optimization technique fine-tunes network parameters to decrease reconstruction error across training pictures. Skip connections connect the input of one layer to the output of the next layer, enhancing training stability and gradient flow. The Adaptive Shrinkage Unit (ASU) trains shrinkage coefficients locally to reduce input picture noise. ASU adaptively determines appropriate shrinkage values for picture components to reduce noise and preserve essential characteristics. Figure 4 shows the typical ADAE construction technique.

Figure 4 illustrates the ADAE. In the following manner, for a given noise image, the ASU produces the shrinkage parameters α represented in Equation 7:


$$\begin{array}{l} \alpha =\sigma \left(Wx+b\right) \end{array}$$ (7)

The sigmoid activation function is denoted by σ, while the weights and biases acquired during training are represented by W and b. Using the noisy image $\hat{x}$ = = Ax, the denoised image $\hat{x}$ is produced by element-wise multiplication with shrinkage parameters. As noise intensity grows across picture segments, shrinkage coefficients adjust, removing noise components. ASU increases network adaptability across datasets and noise levels and reduces dependence on manually established threshold functions, improving predictive powers and efficiency. ADAE's proposed structure contains many essential features,

Encoder: Convolutional layers, which focus on condensing temporal information, are used to learn image representations.

Decoder: Image details are recovered through the use of deconvolution layers during image reconstruction.

Adaptive Shrinkage Unit (ASU): Effectively eliminates noise while maintaining features by learning shrinkage coefficients using local attention methods.

Dropout Regularization: Included after the first convolutional layer to improve the network's durability and flexibility by simulating real-world noise situations.

Shortcut Connection: Increases performance on an array of deep learning tasks by increasing training efficiency, stability, and generalization ability.

#### 3.2.3 Gaussian deep belief network (GDBN)

The Deep Belief Network (DBN) model consists of many layers with different types of neurons. The visible layer is the lowest layer of the neural network and receives pixel-level image input, while the subsequent layers represent the hidden layers. Neurons in two neighboring levels are completely connected. The layers are connected from top to bottom (Sarikaya et al., 2014). In a DBN with m levels, image pixels are inserted into the visible layer, which is the base layer. To determine the probabilities of the previous layer of neurons, the neurons in the top two layers have bidirectional connections, which can be thought of as two levels in the Restricted Boltzmann Machines (RBM) structure. The probability of accessing variables at each layer of the DBN depends on the top-layer parameters and neuron connections. A sigmoid confidence block may be thought of as each layer of the deep confidence network. During Gibbs sampling for image creation, a top-layer restricted Boltzmann machine operates initially, repeatedly balanced (Wei et al., 2019). The energy function is stated as expressed in Equation 8, to reach thermal equilibrium:


$$\begin{array}{lll} \text{E}\left(\text{v},\text{h}\right) & = & -a^{T}v-b^{T}v-v^{T}wh \end{array}$$ (8)

The representations for the visible and hidden units are ν and h, respectively, and the biases of *a* and *b*, the weights between ν and h, and *w*; respectively, on the visible and hidden units. In each hidden layer, the conditionally distributed sampling of variables is repeated once equilibrium is achieved, and the potential value of the subsequent hidden layer is established. Equation 9 is provided for both the hidden and visible layers' combined probability distribution:


$$\text{p}\left(\text{v},\text{h}\right)=\frac{{\text{e}}^{-\text{E}(v,h)}}{\sum_{\text{v}}\sum_{\text{h}}{\text{e}}^{-\text{E}(\text{v},\text{h})}}$$ (9)

It is possible to sample the lower-layer neuron separately, as its value is conditionally independent of the upper-layer neuron's. The following is the sampling procedure. Equation 10 determines that it is probable that neurons in the buried layer may activate.


$$\begin{array}{l} \text{p}\left({\text{h}}_{\text{f}}|\text{v}\right)=\text{σ}\left({\text{b}}_{\text{f}}+\sum_{\text{j}}{\text{w}}_{\text{ij}}{\text{v}}_{\text{i}}\right) \end{array}$$ (10)

Equation 11 gives the chance that the hidden-layer neuron will cause the visible-layer neuron to be active.


$$\begin{array}{l} \text{p}\left({\text{v}}_{\text{i}}|\text{h}\right)=\text{σ}\left({\text{a}}_{\text{i}}+\sum_{\text{j}}{\text{w}}_{\text{ij}}{\text{h}}_{\text{j}}\right) \end{array}$$ (11)

**Figure 5:**
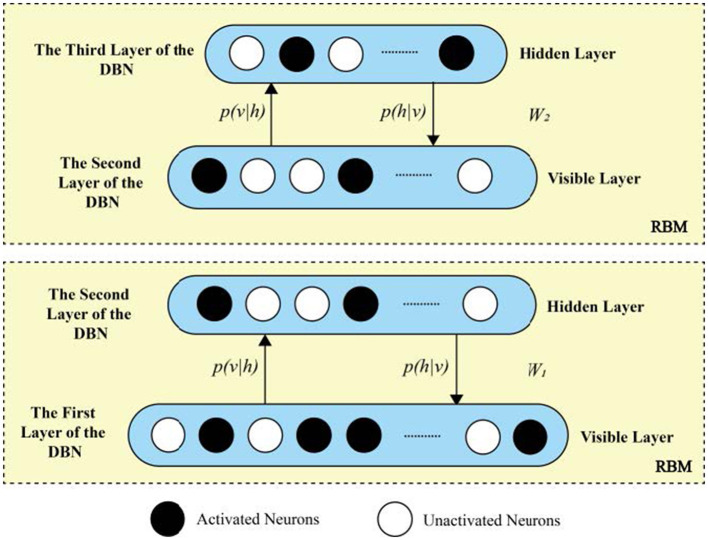
Layer-by-layer pre-training process.

Sigmoid activation function σ is used in this context. The independence of the neurons in the same layer gives the probability density another characteristic. First, ascertain the number of network layers, weights, biases, training set, and learning rate. Following that, set the bias initialization to zero, with a variance of 0.1, and generate random numbers with the weights set to zero to produce a normal distribution mean. The hidden variables at the next layer are then inferred from the training image, and their values and probabilities are determined based on the current layer's hidden variables. This process is repeated until the last layer is reached. The training weight and bias parameters for the final layer are then established using an RBM, after a training image is produced from the second-to-last layer's implicit variables (Wei et al., 2019). Figure 5 depicts the training procedure.

**Figure 6:**
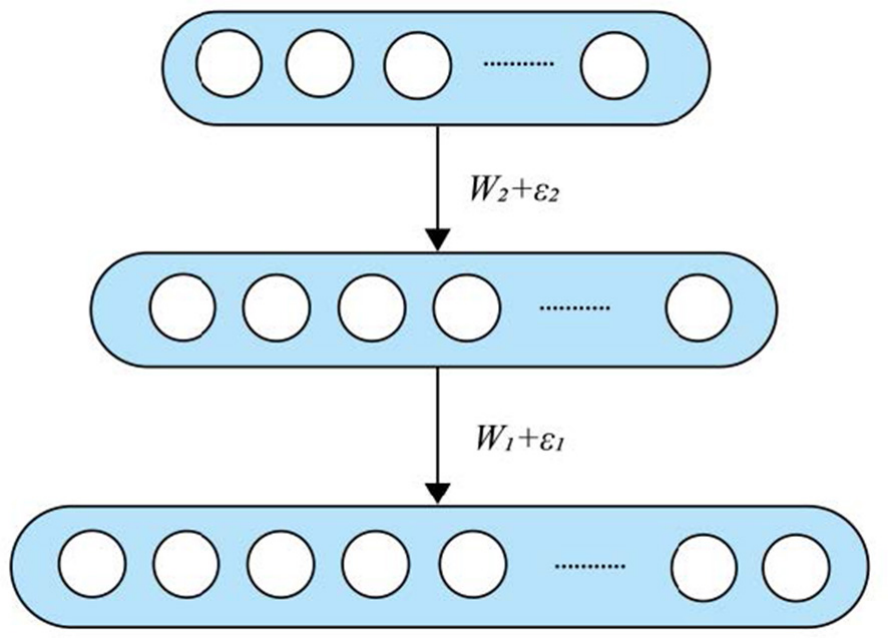
Illustration of the fine-tuning process.

It is possible to describe the connection weights between each pair of layers by combining upward cognitive and downward production matrices. The DBN precisely adjusts the weights to accelerate the model's convergence to a local optimum. Figure 6 shows the fine-tuning procedure. To enhance the upper-layer sample efficiency of the upward cognition weight matrix, the weight matrix must be adjusted. When reaching thermal equilibrium, before computing the upper layer of the RBM by maximizing the upward conditional probability, the cognitive weight matrix is created (Yan et al., 2020).

The RBMs in the hidden layers of a typical DBN are usually binary, enabling the extraction of image features but often at the expense of some information. To combat this, a DBN with Gaussian RBMs (GRBMs) is introduced, creating a Gaussian DBN (GDBN). GRBMs can handle continuous data, making them well-suited for processing image pixel values, which are typically real-valued in practice. Secondly, GRBMs offer a more accurate representation of features, with the ability to better represent real-valued distributions. The latent features produced by GRBMs are generally more precise than those produced by binary RBMs. GRBM can represent the complex structure of images due to its more flexible probability density function. The energy function of a GRBM is expressed as follows Equation 12:


$$\begin{array}{l} \text{E}\left(\text{v},\text{h}\right)=-\overset{{\text{n}}_{\text{v}}}{\underset{\text{i}=1}{\sum }}\frac{{\left({\text{v}}_{\text{i}}-{\text{a}}_{\text{i}}\right)}^{2}}{2\sigma_{\text{i}}^{2}}-\overset{{\text{n}}_{\text{h}}}{\underset{\text{j}=1}{\sum }}\frac{{\left({\text{h}}_{\text{j}}-{\text{b}}_{\text{j}}\right)}^{2}}{2\sigma_{\text{j}}^{2}}\\ \;-\overset{{\text{n}}_{\text{v}}}{\underset{\text{i}=1}{\sum }}\overset{{\text{n}}_{\text{h}}}{\underset{\text{j}=1}{\sum }}\frac{\left({\text{v}}_{\text{i}}-{\text{a}}_{\text{i}}\right)\left({\text{h}}_{\text{j}}-{\text{b}}_{\text{j}}\right)}{\sigma_{\text{i}}\sigma_{\text{j}}}{\text{w}}_{\text{ij}} \end{array}$$ (12)

The visible layer neurons' state vector is represented by ν in this case, whereas the hidden layer (HL) neurons' state vector is shown by *h*. The *i*th hidden and *i*th visible neurons' bias values are denoted by the words *a*_*i*_ and *b*_*j*_, respectively. The aggregate quantity of neurons in the HL and visible layers is indicated by variables *i* and *j*. The noise variances for the *i*th hidden and *i*th visible neurons are given by σ_*i*_^2^ and σ_*j*_^2^, respectively. Finally, *w*_*ij*_ represents how much weight is attached to the connection between the *i*th hidden and *i*th visible neurons. The positive distribution function at the moment of sampling is selected as part of the DBN improvement process, and it is computed as expressed in Equation 13:


$$\begin{array}{l} \text{p}\left({\text{x}}_{\text{i}}=\text{x}|\text{x}/\text{i}\right)=\frac{1}{\sqrt{2\pi {\text{σ}}^{2}}}\exp(\frac{-\left(x-\mu \right)}{2\sigma^{2}}) \end{array}$$ (13)

where σ is 1 and μ is the parameter that was learned by the GRBM network and can be computed from the current state x_i. In GRBM, the explicit layer's conditional probability is given by Equation 14 to the hidden layer:


$$\begin{array}{l} \text{p}\left({\text{v}}_{\text{i}}|\text{h}\right)=\text{N}\left({\text{a}}_{\text{i}}+\underset{\text{j}}{\sum }{\text{w}}_{\text{ij}}{\text{h}}_{\text{j}},1\right) \end{array}$$ (14)

In GRBM, the hidden layer's conditional probability formula for the following Equation 15 is the explicit layer:


$$\begin{array}{l} \text{p}\left({\text{h}}_{\text{j}}|\text{v}\right)=\text{N}\left({\text{b}}_{\text{j}}+\underset{\text{j}}{\sum }{\text{w}}_{\text{ij}}{\text{v}}_{\text{i}},1\right) \end{array}$$ (15)

#### 3.2.4 Ensemble of denoising models

After training on the training image dataset, the models are assessed on the verification image dataset, and the weighted average of each model's PSNR is used to construct the final denoised image. Regarding the weights, each model's performance on the images is evaluated individually, and the resulting results are utilized to determine the models' appropriate ensemble ratio.

### 3.3 Image augmentation

Data augmentation is a solution to the problem of data imbalance. Techniques were applied. In the proposed framework, augmented versions of the original image slices were created using operations such as rotation, translation, gamma adjustment, scaling, and affine transformations. This is to alleviate potential overfitting during CNN training and to offset differences in the input images. In this step, augmentation techniques will be used to expand the dataset's size. The model cannot learn fixed spatial patterns when the brain's location in the pictures is dramatically changed. The MRI pre-processing protocol includes the following steps:

Rotation: Rotated images at 15 angles without cropping, as the disease might not be completely visible in the cropped image.

Translation: Image translation is the process of transforming an image representation into another. The objective is to learn how the input and output images are transformed.

Gamma Correction: Gamma correction is a non-linear operation that adjusts the brightness and contrast of an image to compensate for the non-linearity of image-capture devices. It's applied to each picture pixel, and the gamma value shapes the correction curve. MRI images are pre-processed and improved using adaptive gamma correction (AGC). Parameters change dynamically with picture data.

Scaling: The MRI image processing uses scaling to standardize intensity scales so that comparable intensities yield equivalent tissue interpretations. This matters because standard MRI scans employ arbitrary scales, making measurement difficult. The Arbitrary Scale Super-Resolution Diffusion Model (ASSRDM) generates high-quality medical pictures using a denoising diffusion probabilistic framework and an implicit neural representation. Its scaling factor controls picture resolution and the emphasis on output detail.

Affine Transformation: One of the most frequent linear transformations, the affine transformation, treats the picture as rigid. This class provides uniform and non-uniform scaling, rotation, shearing, and reflection for all rigid body movements. It has 12 degrees of freedom in three dimensions.

### 3.4 Image segmentation

The OTSU-Levy Grasshopper Optimization Algorithm determines the segmentation Region of Interest (RoI) from MRI images. OTSU thresholding improves segmentation by comparing the MRI scan mean pixel intensity to a threshold value. Initially, this method divides the MRI images into two distinct areas: the dark region *T*_1_ and the bright region *T*_0_ (Goh et al., 2018; Tan et al., 2021). The mathematical expressions for these regions are presented in Equations 16, 17,


$$\begin{array}{lll} T_{1} & = & \left\{t,t+1,\ldots .,l-1,l\right\} \end{array}$$ (16)

$$\begin{array}{lll} T_{0} & = & \left\{0,1,\ldots .,t\right\} \end{array}$$ (17)

The variables *t* and *l* represent the histogram bins and the threshold value, respectively. Choosing the optimal threshold is crucial for effectively separating overlapping classes. Minimizing within-class variance provides the best value in standard OTSU thresholding, which depends on the probability distributions of the various groups *d*(*i*). This relationship is mathematically defined in Equation 18.


$$\begin{array}{l} d\left(i\right)=\frac{number\left\{\left(r,c\right)|image\left(r,c\right)=i\right\}}{\left(r,c\right)} \end{array}$$ (18)

where (r, c) stands for the collected MRI images' rows and columns. In the MRI images, band f represents the foreground and background areas, and the variance $\sigma_{b}^{2}\left(t\right)$ and $\sigma_{f}^{2}\left(t\right)$, mean μ_*b*_(*t*) and μ_*f*_(*t*), and weight *w*_*b*_(*t*) and *w*_*f*_(*t*) of the areas that are radiant and dark *T*_0_ and *T*_1_ are determined using Equations 19, 20.


$$\begin{array}{l} w_{b}\left(t\right)=\overset{t}{\underset{i=1}{\sum }}d\left(i\right),\;\mu_{b}\left(t\right)=\frac{\sum_{i=1}^{t}i\times d\left(i\right)}{w_{b}\left(t\right)},\;\sigma_{b}^{2}\left(t\right)\\ \;=\frac{\sum_{i=1}^{t}{\left(i-\mu_{b}\left(t\right)\right)}^{2}\times d\left(i\right)}{w_{b}\left(t\right)} \end{array}$$ (19)

$$\begin{array}{l} w_{f}\left(t\right)=\overset{l}{\underset{i=t+1}{\sum }}d\left(i\right),\;{\text{μ}}_{\text{f}}\left(\text{t}\right)=\frac{\sum_{\text{i}=\text{t}+1}^{\text{l}}\text{i}\times \text{d}\left(\text{i}\right)}{{\text{w}}_{\text{f}}\left(\text{t}\right)},\;{\text{σ}}_{\text{f}}^{2}\left(\text{t}\right)\\ \;=\frac{\sum_{\text{i}=\text{t}+1}^{\text{l}}{\left(\text{i}-{\text{μ}}_{\text{f}}\left(\text{t}\right)\right)}^{2}\times \text{d}\left(\text{i}\right)}{{\text{w}}_{\text{f}}\left(\text{t}\right)} \end{array}$$ (20)

With low class variance $\sigma_{w}^{2}$, the OTSU thresholding approach determines the optimal threshold. Equation 21 represents the low-class variance $\sigma_{w}^{2}$. To obtain the optimal threshold value with minimal execution time, LGOA is combined with the OTSU thresholding approach.


$$\begin{array}{l} {\text{σ}}_{\text{w}}^{2}={\text{w}}_{\text{b}}\left(\text{t}\right)\times {\text{σ}}_{\text{b}}^{2}\left(\text{t}\right)+{\text{w}}_{\text{f}}\left(\text{t}\right)\times {\text{σ}}_{\text{f}}^{2}\left(\text{t}\right) \end{array}$$ (21)

The swarm's grasshoppers' positions serve as potential thresholds for the OTSU and the Grasshopper Optimization Algorithm (GOA). Social interaction, gravity, and wind advection are the three basic elements that affect grasshopper movement. Equation 22 represents the threshold selection of the ith grasshopper as *X*_*i*_ (Meraihi et al., 2021),


$$\begin{array}{l} {\text{X}}_{\text{i}}={\text{S}}_{\text{i}}+{\text{G}}_{\text{i}}+{\text{A}}_{\text{i}} \end{array}$$ (22)

Here, *A*_*i*_ is wind advection, *S*_*i*_ is grasshopper social interaction, *X*_*i*_ is the ith grasshopper, and *G*_*i*_ is the gravitational force acting on the position for threshold selection. The social interaction has been calculated using Equation 23:


$$\begin{array}{l} {\text{X}}_{\text{i}}={\text{r}}_{1}{\text{S}}_{\text{i}}+{\text{r}}_{2}{\text{G}}_{\text{i}}+{\text{r}}_{3}{\text{A}}_{\text{i}} \end{array}$$ (23)

where the random integers in the range are *r*_1_, *r*_2_, *r*_3_∈[0, 1]. Equations 24–27 for social interaction *S*_*i*_ are as follows:


$$\begin{array}{lll} {\text{S}}_{\text{i}} & = & \overset{\text{N}}{\underset{\begin{array}{c} \text{j}=1\\ \text{j}\neq 1 \end{array}}{\sum }}\text{s}\left({\text{d}}_{\text{ij}}\right){\hat{\text{d}}}_{\text{ij}} \end{array}$$ (24)

$$\begin{array}{lll} {\text{d}}_{\text{ij}} & = & \left|{\text{X}}_{\text{j}}--{\text{X}}_{\text{i}}\right| \end{array}$$ (25)

$$\begin{array}{lll} {\hat{\text{d}}}_{\text{ij}} & = & \frac{{\text{P}}_{\text{j}}-{\text{P}}_{\text{i}}}{{\text{d}}_{\text{ij}}} \end{array}$$ (26)

$$\begin{array}{lll} \text{s}\left(\text{r}\right) & = & \text{f}{\text{e}}^{\frac{-\text{r}}{\text{l}}}-{\text{e}}^{-\text{r}}\; \end{array}$$ (27)

where *N* is denoted as the number of grasshoppers based on the threshold selection, *d*_*ij*_ is denoted as the Euclidean distance between the ith and the jth grasshopper, ${\hat{d}}_{ij}$is a unit vector, and *s* is denoted as the social forces. The gravitational force of a grasshopper *G*_*i*_ by Equation 28:


$$\begin{array}{lll} {\text{G}}_{\text{i}} & = & {\text{gê}}_{\text{g}} \end{array}$$ (28)

where *g* is represented as the direction of the center of the unit vector, Earth is the gravitational constant, and ê_*g*_. Equation 29 gives the wind advection *A*_*i*_:


$$\begin{array}{lll} {\text{A}}_{\text{i}} & = & {\text{uê}}_{\text{w}} \end{array}$$ (29)

where ê_*w*_is a vector representing the wind direction and the drift constant. Then, it can be expanded by Equation 30:


$$\begin{array}{l} {\text{X}}_{\text{i}}=\overset{\text{N}}{\underset{\begin{array}{c} \text{j}=1\\ \text{j}\neq 1 \end{array}}{\sum }}\text{s}\left(\left|{\text{X}}_{\text{j}}-{\text{X}}_{\text{i}}\right|\right)\frac{{\text{X}}_{\text{j}}-{\text{X}}_{\text{i}}}{{\text{d}}_{\text{ij}}}-{\text{gê}}_{\text{g}}+{\text{uê}}_{\text{w}} \end{array}$$ (30)

Grasshopper in the *A* is always toward the threshold solution ${\hat{T}}_{d}$. It is *d*. It is constructed by Equation 31:


$$\begin{array}{l} {\text{X}}_{\text{i}}^{\text{d}}=\text{ c}\left(\overset{\text{N}}{\underset{\begin{array}{c} \text{j}=1\\ \text{j}\neq 1 \end{array}}{\sum }}\text{c}\frac{\text{u}{\text{b}}_{\text{d}}-\text{l}{\text{b}}_{\text{d}}}{2}\text{s}\left(\left|{\text{X}}_{\text{j}}^{\text{d}}-{\text{X}}_{\text{i}}^{\text{d}}\right|\right)\frac{{\text{X}}_{\text{j}}-{\text{X}}_{\text{i}}}{{\text{d}}_{\text{ij}}}\right)+{\hat{\text{T}}}_{\text{d}} \end{array}$$ (31)

where *ub*_*d*_ and *lb*_*d*_ is denoted as the threshold selection upper and lower boundaries in the dth dimension. Equation 32 uses parameter c to minimize the area among grasshoppers to the number of phases,


$$\begin{array}{l} \text{c}={\text{c}}_{\max}-\text{l}\frac{{\text{c}}_{\max}-{\text{c}}_{\min}}{\text{L}} \end{array}$$ (32)

The values of *c*_max_ and *c*_min_ indicate the current iteration's maximum and minimum c values, where L is the maximum. The positions of other grasshoppers in the swarm, the global best position, and the grasshopper's present position are each considered by the grasshopper to update its position.

**L**evy Flight (LF): A stochastic, non-Gaussian walk technique is used for threshold selection. LF is accomplished by the use of Equation 33:


$$\begin{array}{l} \text{Levy}\left(\text{β}\right)\sim \text{u}={\text{t}}^{-1-\text{β}},0<\text{β}\leq 2 \end{array}$$ (33)

β is denoted as the parameter to modify stability, using the Levy index. It has been calculated by Equation 34:


$$\begin{array}{l} \text{Levy}\left(\text{β}\right)\sim \frac{\text{φ}\times \text{μ}}{|\text{v}{|}^{\frac{1}{\text{β}}}} \end{array}$$ (34)

where μ is a typical Gamma function, β = 1.5, ϕ is determined by Equation 35, and μ and v are the two standard normal distributions.


$$\begin{array}{l} \text{φ}={\left[\frac{\text{Γ}\left(1+\text{β}\right)\times \sin\left(\text{π}\times \frac{\text{β}}{2}\right)}{\text{Γ}\left(\left(1\times \frac{\text{β}}{2}\right)\times \text{β}\times 2^{\frac{\text{β}-1}{2}}\right)}\right]}^{\frac{1}{\text{β}}} \end{array}$$ (35)

**Figure 7:**
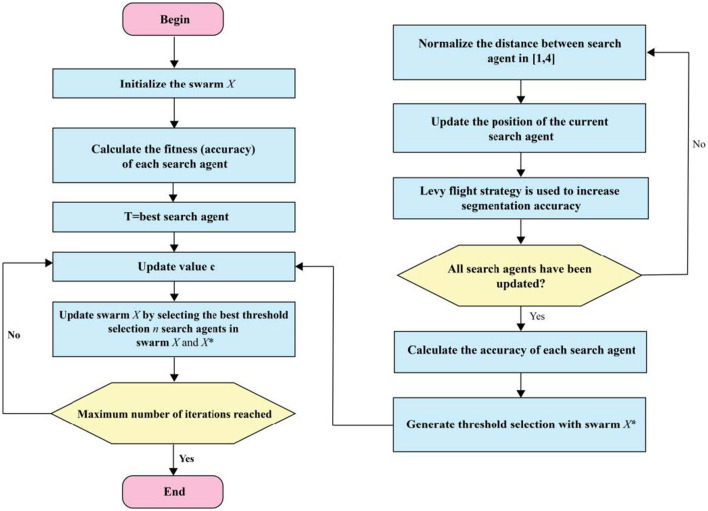
Flowchart of LGOA.

LF distribution is formulated by Equation 36:


$$\begin{array}{l} {\text{X}}_{\text{i}}^{\text{Levy}}={\text{X}}_{\text{i}}+\text{r}⊕\text{levy}\left(\text{β}\right) \end{array}$$ (36)

where $X_{i}^{Levy}$, ⊕ is the dot product, and in [0,1], the random vector r denotes the new threshold position of the ith search agent *X*_*i*_ following Equations 37, 38 are utilized to develop a new threshold selection when the position of the ith grasshopper, *X*_*i*_, has been modified.


$$\begin{array}{lll} {\text{X}}_{\text{i}}^{\text{Levy}} & = & {\text{X}}_{\text{i}}^{*}+\text{rand}\left(\text{d}\right)⊕\text{levy}\left(\text{β}\right) \end{array}$$ (37)

$$\begin{array}{lll} {\text{X}}_{\text{i}}^{\text{t}+1} & = & \{\begin{array}{c} {\text{X}}_{\text{i}}^{\text{Levy}}\text{ fitness}\left({\text{X}}_{\text{i}}^{\text{Levy}}\right)>\text{fitness}\left({\text{X}}_{\text{i}}^{*}\right)\\ X_{i}^{*}\text{ otherwise} \end{array} \end{array}$$ (38)

where rand(d) is a d-dimensional random vector in [0,1] and $X_{i}^{*}$ is the ith grasshopper's new key position upon updating. To determine the appropriate threshold from the images, the LF mechanism has a high probability of converging to a local optimum. Algorithm 1 provides the LGOA method and Figure 7.

Algorithm 1 Pseudo-code of LGOA

1. Determine the Grasshoppers' starting population
   *X*_*i*_(i = 1 to n)
2. Initialize *c*_min_, *c*_max_ minimum and maximum number
   of iterations
3. For each search agent (*X*_*i*_, determine the
   fitness *f*(*X*_*i*_)
4. T = Best thershold for
5. While (l < *L*) do
    5.1. Update *c* using Equation 32
    5.2. For *i* = 1 *to N*, each search agent do
      5.2.1. The distance between grasshoppers in
             the range should be normalized [1, 4].
      5.2.2. Improve the existing search agent's
             threshold position using Equation 31
      5.2.3. Levy flight strategy using
             Equations 33, 34 and
      5.2.4. LF mechanism is used to generate a new
             threshold selection by Equations 37, 38
    5.3. End for
    5.4. Update *T* if a better threshold solution is
         available
5. Increase *l* = *l*+ 1 in the iteration
6. End while
7. Return the threshold solution *T*
8. End

### 3.5 Cross-validation

A common method for evaluating (CV) cross-validation evaluates deep learning models' performance. There are many subgroups within the dataset using this technique: some are used for training models and others for assessing performance. In deep learning, k-fold CV is often used (Wong and Yeh, 2019). The dataset has been divided into k “folds” for k-fold CV, each of the same size. The model uses a different fold as the verification set for each of its k training stages, with the remaining folds being utilized for training (Jung, 2018). Averaging the model's performance across k iterations determines its overall performance. This method aims to expose the model to various training and test splits, thereby optimizing its performance for Alzheimer's disease (AD) diagnosis.

### 3.6 Feature extraction and classification

CNNs can handle enormous picture datasets and perform well in detection. Feature extraction from raw photos produces meaningful data for learning models. This model then classifies or predicts new pictures using extracted characteristics. Most feature extraction uses image-trained deep neural networks. These networks train on large datasets to learn high-level abstractions from raw images, extracting properties. A CNN has numerous layers, including FC, max pooling, and CL, each intended to extract features.

Convolution layer (CL): The convolutional layer uses a fixed-size kernel to produce feature maps from the input picture. This kernel scans the picture and performs element-wise multiplication to capture distinct aspects, which is sent to the next layer. CNNs have many hidden layers that recognize distinct characteristics. Typically, the initial layer captures simple or low-level features, and its output is passed to the next layer, which detects more complex patterns. Each hidden layer is assigned an activation function, and all layers use the same one.

Pooling layer: A feature map is produced through average pooling, which computes values above the threshold generally using Equations 39, 40:


$$\begin{array}{lll} \text{Output of Width} & = & \;\frac{\text{W}-\text{F}+2\text{P}}{\text{S}}+1 \end{array}$$ (39)

$$\begin{array}{lll} \text{Output of height} & = & \;\frac{\text{H}-\text{F}+2\text{P}}{\text{S}}+1 \end{array}$$ (40)

The feature map's width is denoted by W, its height by H, its filter size by F, its padding value (if non-zero) by P, and its stride value by S.

Fully connected layer: Each neuron in a fully connected layer is connected to all neurons in the layer below. This layer creates a one-dimensional vector from the multi-dimensional feature map. In most deep learning models, the final layers are fully connected and aggregate information from earlier layers to produce the final prediction. Two neurons are used for binary categorization, while the output layer's neuron count equals the number of classes in multi-class classification.

VGG16 model: A fixed-size (64 × 64) input image is used by the VGG16 model and is trained on an MRI dataset. In this approach, the first 16 layers are kept unchanged (frozen), while only the final convolutional and pooling layers are retrained. Transfer learning involves training a model on smaller, different datasets while freezing certain layers to preserve previously learned knowledge. The convolutional and pooling layers are frozen to preserve feature extraction consistency, and a new classification layer is trained on MRI data. The initial few layers correctly capture basic, broad characteristics; thus, they are frozen, while deeper layers that recognize more complicated patterns are trained on dataset features. The transfer learning approach of fine-tuning involves not freezing all convolutional and pooling layers. Unfreeze and retrain the last convolutional and pooling layers to match the new data. Image enhancement includes 15 degrees of rotation, 0.2 crop, 0.2 zoom, and 0.1 modifications to width and height. On MRI scans, Adam, Adadelta, RMSprop, Adagrad, and SGD optimizers assess the model's performance.

## 4 Experiments and results

In this section, experiments are conducted on an Intel i7-6700 CPU running at 3.4 GHz with 8GB of RAM. Existing approaches and the proposed system were implemented in MATLAB R2022a. In recent work, the confusion matrix is often used to evaluate models because it is a useful measure of classification performance and can be applied regardless of data distribution or class relationships. Image quality is measured using denoising techniques to estimate PSNR, MSE, and SSIM. In classification models, the model is evaluated using a confusion matrix that includes TP, TN, FP, and FN (Jung, 2018). Various performance indicators such as SEN, SPC, PPV, F1, ACC, and AUC are then computed using these four outcomes. Furthermore, three metrics like DSC, Pixel Accuracy (PA), and Intersection over Union (IoU) are used to analyze segmentation performance:

PSNR is calculated using Equations 41, 42:


$$\begin{array}{lll} \text{PSNR} & = & 10\log_{10}{\left(\frac{2^{\text{n}}}{\text{MSE}}\right)}^{2} \end{array}$$ (41)

$$\begin{array}{lll} \text{MSE} & = & \frac{1}{\text{H}\times \text{W}}\overset{\text{H}}{\underset{\text{j}=1}{\sum }}\overset{\text{W}}{\underset{\text{k}=1}{\sum }}{\left(\text{M}\left(\text{j},\text{k}\right)-\text{N}\left(\text{j},\text{k}\right)\right)}^{2} \end{array}$$ (42)

where j and k indicate individual pixels, with M and N representing the expected and actual values, respectively. The value of the parameter n, while H and W stand for the image's height and breadth. One tool for evaluating image similarity is the SSIM. Luminance (brightness), contrast, and structural information are the three criteria to consider when evaluating this similarity. The standard deviation represents contrast, the mean value determines brightness, and covariance quantifies structural similarity. This is used to calculate SSIM as expressed in Equation 43:


$$\begin{array}{l} \text{SSIM}\left(\text{M},\text{N}\right)=\frac{\left(2{\text{μ}}_{\text{m}}{\text{μ}}_{\text{n}}+{\text{c}}_{1}\right)\left(2{\text{σ}}_{\text{mn}}+{\text{c}}_{1}\right)}{\left({\text{μ}}_{\text{m}}^{2}+{\text{μ}}_{\text{n}}^{2}+{\text{c}}_{1}\right)\left({\text{σ}}_{\text{m}}^{2}+{\text{σ}}_{\text{n}}^{2}+{\text{c}}_{2}\right)} \end{array}$$ (43)

Here, μ_*m*_ and μ_*n*_ represent the means of images M and N. Image M's variance is represented by $\sigma_{m}^{2}$, while image N's variance is represented by by $\sigma_{n}^{2}$. Covariance between images M and N is represented by Stability constants *c*_1_ and *c*_2_ are used. A higher SSIM value indicates greater similarity; it ranges from 0 to 1. The two images are identical when the SSIM is 1.

During the segmentation stage, the Dice Similarity Coefficient (DSC) evaluates how closely the two masks resemble one another, while the IoU measures the anticipated-ground-truth overlap between masks or bounding boxes. Intersection over Union (IoU), also known as the Jaccard Index, is a widely accepted evaluation metric for image segmentation and object detection. It measures the overlap between the predicted and ground-truth regions. Equations 44 and 45 are used to calculate IoU and DSC, where PS and GS represent the pixels in the predicted and actual segmentations, respectively. Moreover, the ratio of incorrectly categorized pixels in a class (*pixels*)*l*_*i*_) to all pixels in that class (*pixell*_*ij*_) is known as Pixel Accuracy (PA). Classes are represented by the variable K. The formula for PA is provided in Equation 46.


$$\begin{array}{lll} \text{Intersection over Union}\left(\text{IoU}\right)\left(\text{PS},\text{GS}\right) & = & \;\frac{\left|\text{PS}\cap \text{GS}\right|}{\left|\text{PS}\cup \text{GS}\right|} \end{array}$$ (44)

$$\begin{array}{lll} \text{DSC}\left(\text{PS},\text{GS}\right) & = & \;\frac{2\times |\text{PS}\cap \text{GS}|}{\left|\text{PS}|+|\text{GS}\right|} \end{array}$$ (45)

$$\begin{array}{lll} \text{PA} & = & \frac{\sum_{\text{i}=0}^{\text{K}}\text{pixe}{\text{l}}_{\text{i}}}{\sum_{\text{i}=0}^{\text{K}}\sum_{\text{j}=0}^{\text{K}}\text{pixe}{\text{l}}_{\text{ij}}} \end{array}$$ (46)

where ⋂ is denoted as the intersection area, ∪ and is denoted as the union area.

AUC: Area Under the Receiver Operating Characteristic Curve (AUC) is a widely used, threshold-independent performance metric for evaluating classification models. It represents the probability that a classifier will correctly rank a randomly chosen positive instance higher than a randomly chosen negative instance.

Sensitivity (Recall) (SEN)/True Positive Rate (TPR): The ratio of positive samples to all negative samples is referred to as the SEN or True Positive Rate (TPR). TPR is expressed as in Equation 47:


$$\begin{array}{l} \text{Sensitivity}\left(\text{Recall}\right)\left(\text{SEN}\right)=\;\frac{\text{Tp}}{\text{Tp}+\text{Fn}}\times \;100 \end{array}$$ (47)

Specificity (SPC): SPC is the proportion of samples anticipated to be negative out of all the samples that Equation 48 finds to be negative.


$$\begin{array}{l} \text{Specificity }\left(\text{SPC}\right)=\;\frac{\text{Tn}}{\text{Tn}+\text{Fp}}\times \;100 \end{array}$$ (48)

Precision: The number of samples that were really and anticipated to be positive among all the samples that Equation 49 predicted to be positive is referred to as precision, or positive predictive value.


$$\begin{array}{l} \text{Precision }\left(\text{PPV}\right)=\;\frac{\text{Tp}}{\text{Tp}+\text{Fp}}\times \;100 \end{array}$$ (49)

F1-score: The harmonious F1-score is the average of accuracy and recall. To explain it, Equation 50 is given as


$$\begin{array}{l} \text{F}1-\text{score}=\;\frac{2\;*\text{ Tp}}{2\;*\text{ Tp}+\text{Fp}+\text{Fn}}\times \;100 \end{array}$$ (50)

**ACC**: Equation 51 defines accuracy as the proportion of numbers predicted indicated to all predictions.


$$\begin{array}{l} \text{ACC}=\;\frac{\text{Tp}+\text{Tn}}{\text{Tp}+\text{Fp}+\text{Fn}+\text{Tn}}\times \;100 \end{array}$$ (51)

**Figure 8:**
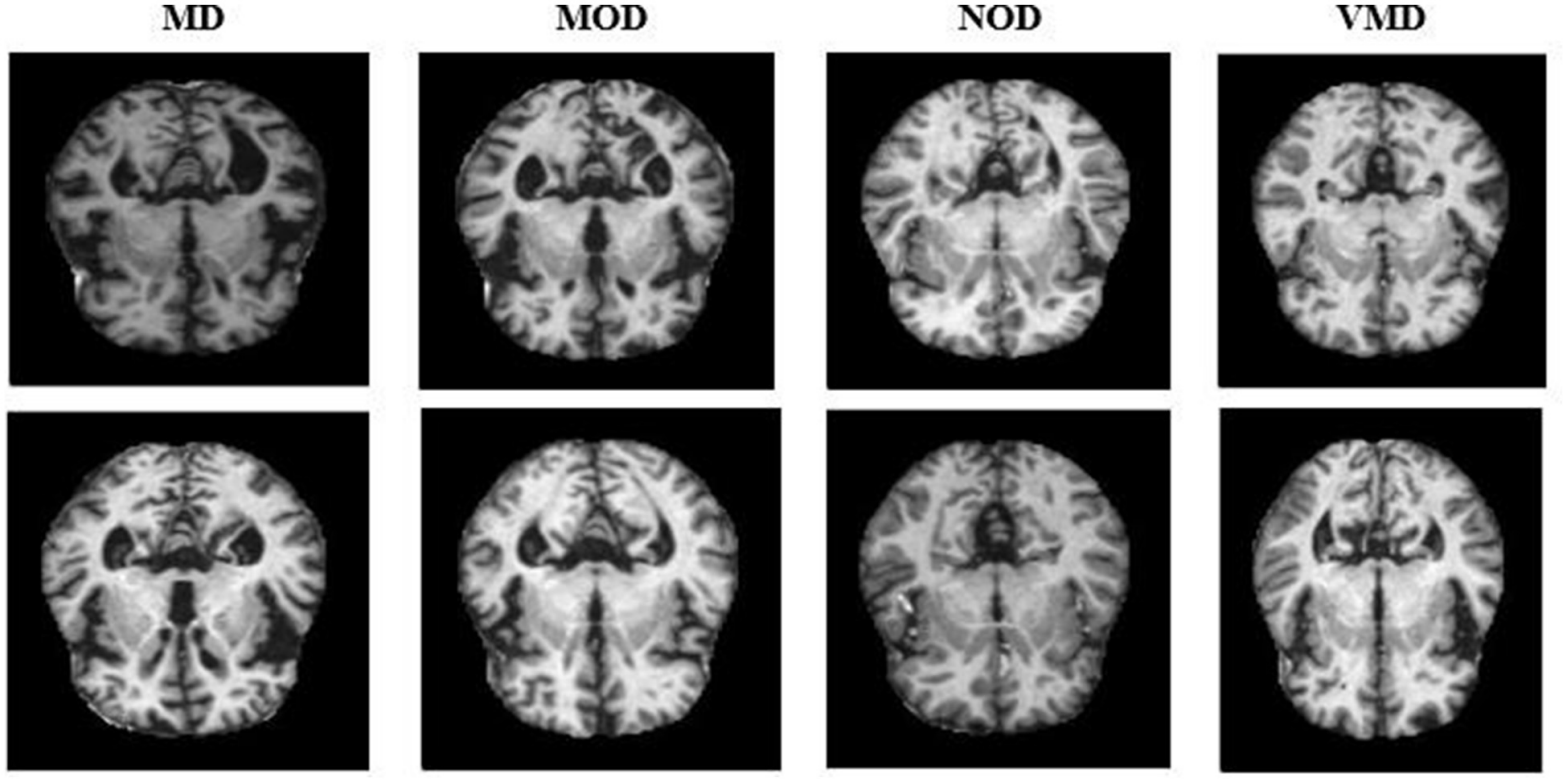
Input image samples.

Figure 8 shows sample images from the input dataset. Table 2 explains the results gathered from the OTSU-LGOA approach and the comparative methodologies. Table 2 makes it explicit that the EDLD method outperforms others in PSNR, MSE, and SSIM.

**Table 2:** Quality metrics comparison vs. image denoising methods.

Methods	PSNR (dB)	MSE	SSIM
DnCNN	33.92	10.74	0.8465
SCNN	35.75	9.56	0.8578
AGCNN	37.27	7.98	0.8704
ADAE	39.49	6.65	0.8866
GDBN	40.75	5.34	0.9048
EDLD	42.46	4.12	0.9276

**Figure 9:**
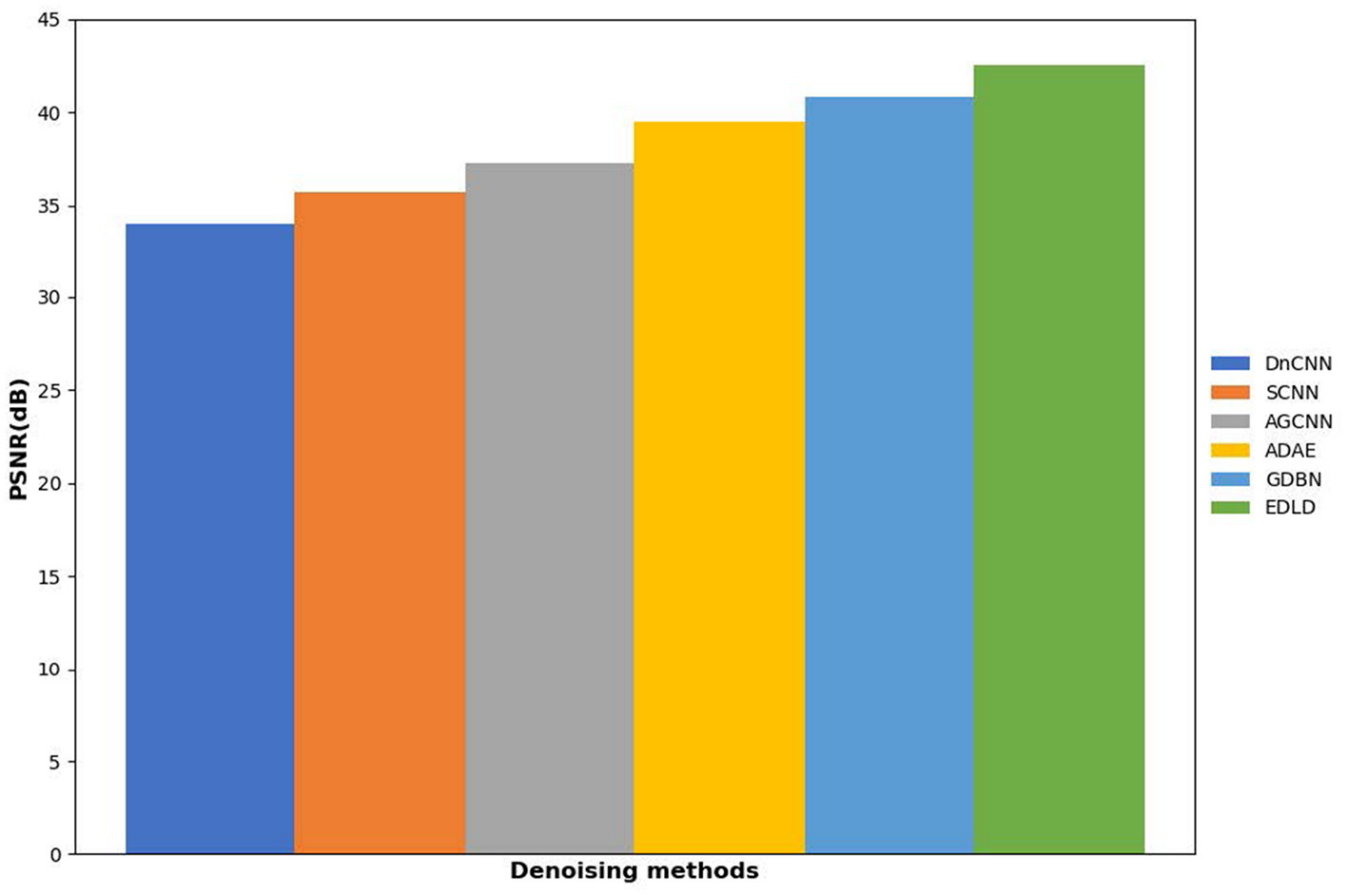
PSNR results comparison of denoising methods.

**Figure 10:**
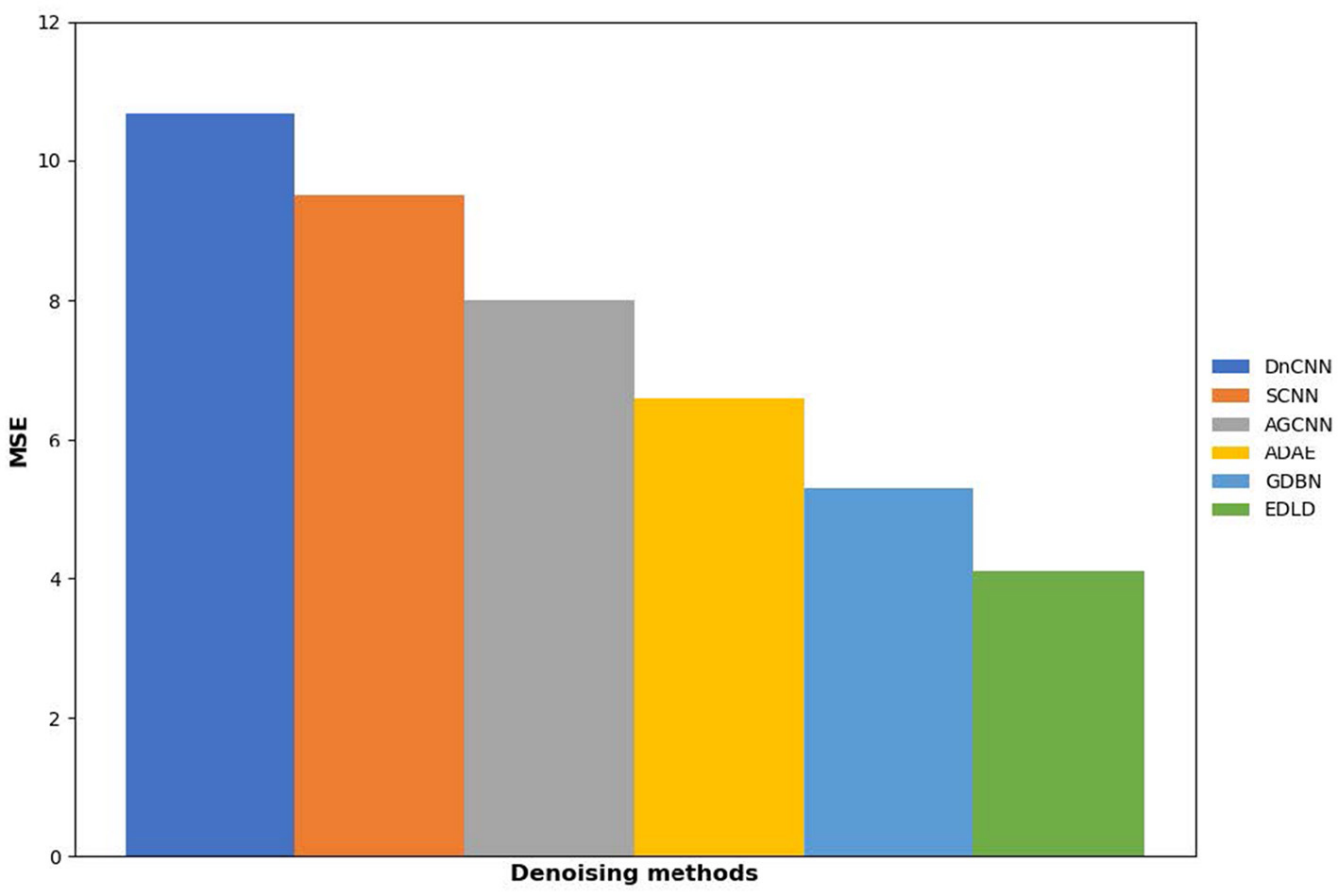
MSE results comparison of denoising methods.

Figure 9 shows the PSNR comparisons for DnCNN, SCNN, AGCNN, ADAE, GDBN, and EDLD. The EDLD method achieves the highest PSNR of 42.46 dB, while DnCNN, SCNN, AGCNN, ADAE, and GDBN achieve 33.92, 35.75, 37.27, 39.49, and 40.75 dB, respectively. The proposed system has 8.54, 6.71, 5.19, 2.97, and 1.71 dB (see Table 2). Figure 10 shows the MSE comparisons for the DnCNN, SCNN, AGCNN, ADAE, GDBN, and EDLD methods. The EDLD system has the lowest score of 4.12, while DnCNN, SCNN, AGCNN, ADAE, and GDBN give scores of 10.74, 9.56, 7.98, 6.65, and 5.34, respectively. The proposed system has 6.62, 5.44, 3.86, 2.53, and 1.22 (see Table 2).

**Figure 11:**
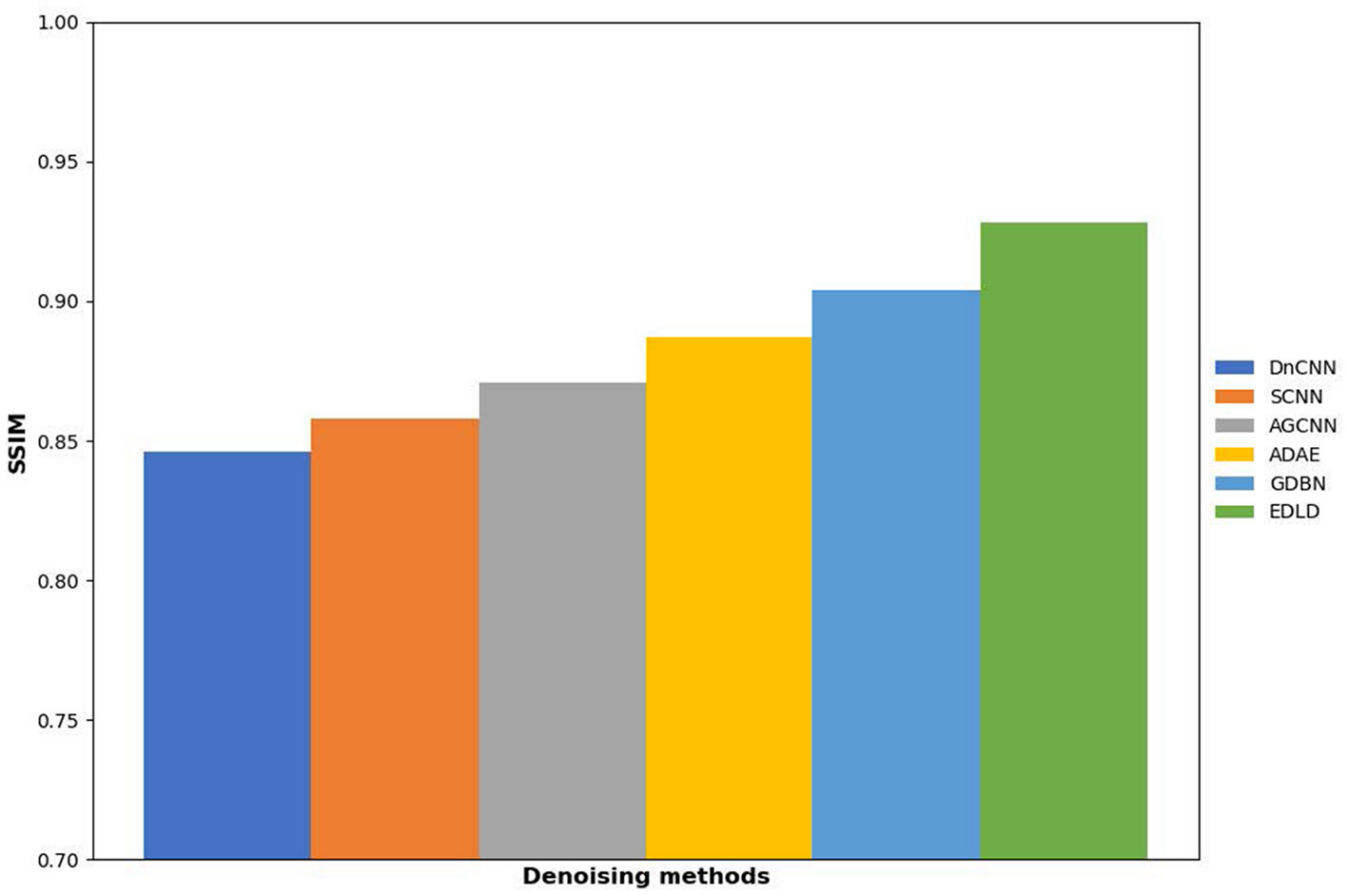
SSIM results comparison of denoising methods.

SSIM comparisons of DnCNN, SCNN, AGCNN, ADAE, GDBN, and EDLD methods are illustrated in Figure 11. The EDLD method has the highest SSIM of 0.9276, while DnCNN, SCNN, AGCNN, ADAE, and GDBN achieve 0.8465, 0.8578, 0.8704, 0.8866, and 0.9048, respectively. The proposed system has 8.74, 7.52, 6.17, 4.42, and 2.46% (see Table 2).

**Figure 12:**
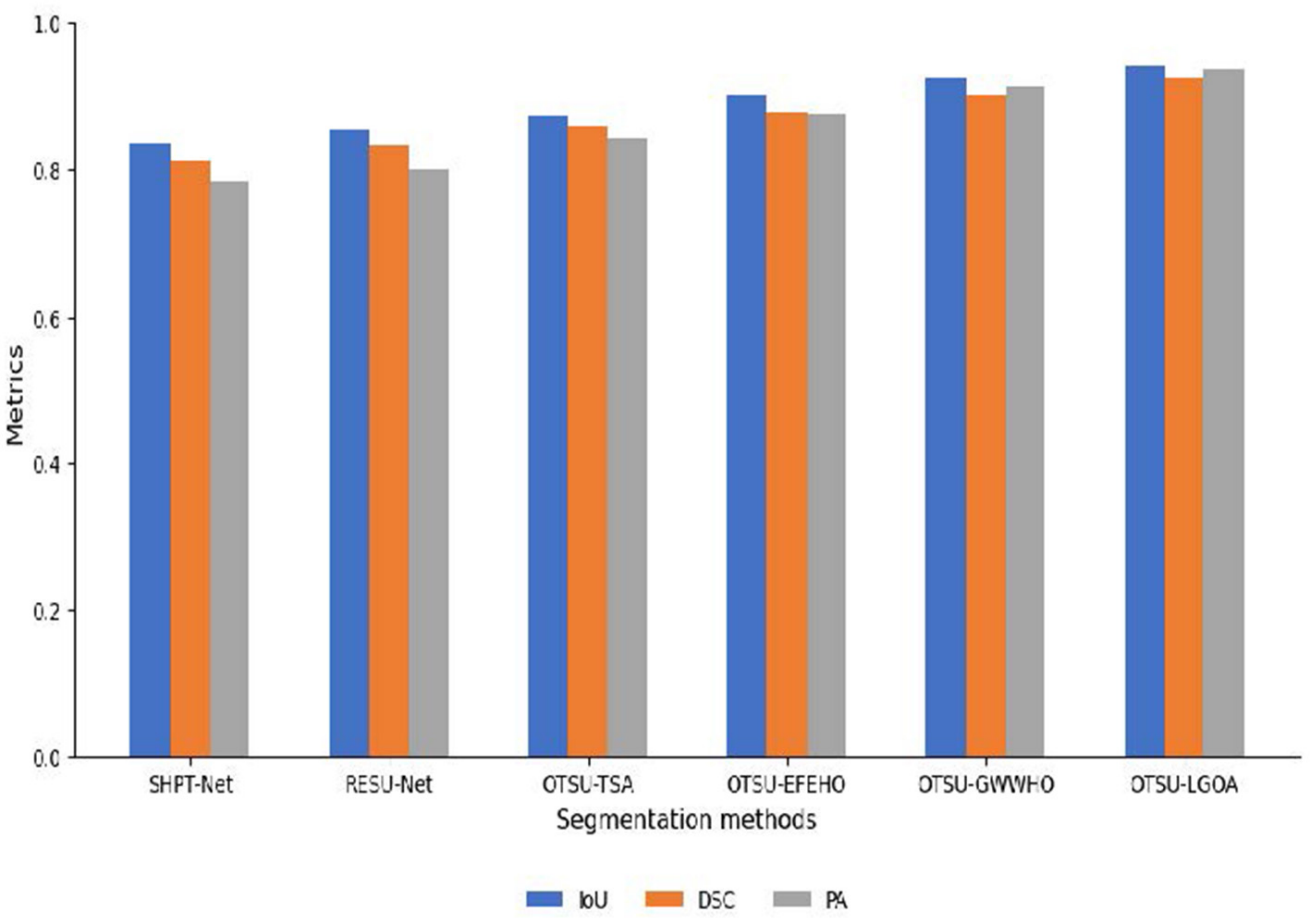
Results comparison of segmentation methods.

Figure 12 compares the effectiveness of the recommended segmentation technique (OTSU-LGOA) with other segmentation techniques in terms of IoU, DSC, and PA on the dataset, such as OTSU-GWWHO, OTSU-EFEHO, OTSU-TSA, RESU-Net, and SHPT-Net. According to Table 3, the suggested approach obtained IoU, DSC, and PA values of 0.9417, 0.9249, and 0.9368, respectively. Other methods, such as SHPT-Net, RESU-Net, OTSU-TSA, OTSU-EFEHO, and OTSU-GWWHO, give the lowest IoU of 0.8344, 0.8563, 0.8716, 0.9041, and 0.9258 (see Table 3). SHPT-Net, RESU-Net, OTSU-TSA, OTSU-EFEHO, and OTSU-GWWHO give the lowest DSC of 0.8116, 0.8342, 0.8575, 0.8784, and 0.9027 (see Table 3). SHPT-Net, RESU-Net, OTSU-TSA, OTSU-EFEHO, and OTSU-GWWHO give the lowest PA of 0.7847, 0.8015, 0.8434, 0.8765, and 0.9149 (see Table 3). An exhaustive search strategy is used for region segmentation in the traditional OTSU thresholding technique, which is computationally intensive and time-consuming. To optimize the threshold value, LGOA is included in the traditional OTSU thresholding approach.

**Table 3:** Segmentation methods vs. evaluation metrics.

Methods	IoU	DSC	PA
SHPT-Net	0.8344	0.8116	0.7847
RESU-Net	0.8563	0.8342	0.8015
OTSU-TSA	0.8716	0.8575	0.8434
OTSU-EFEHO	0.9041	0.8784	0.8765
OTSU Gaussian Weight Wild Horse Optimization (OTSU-GWWHO)	0.9258	0.9027	0.9149
OTSU-LGOA	0.9417	0.9249	0.9368

**Figure 13:**
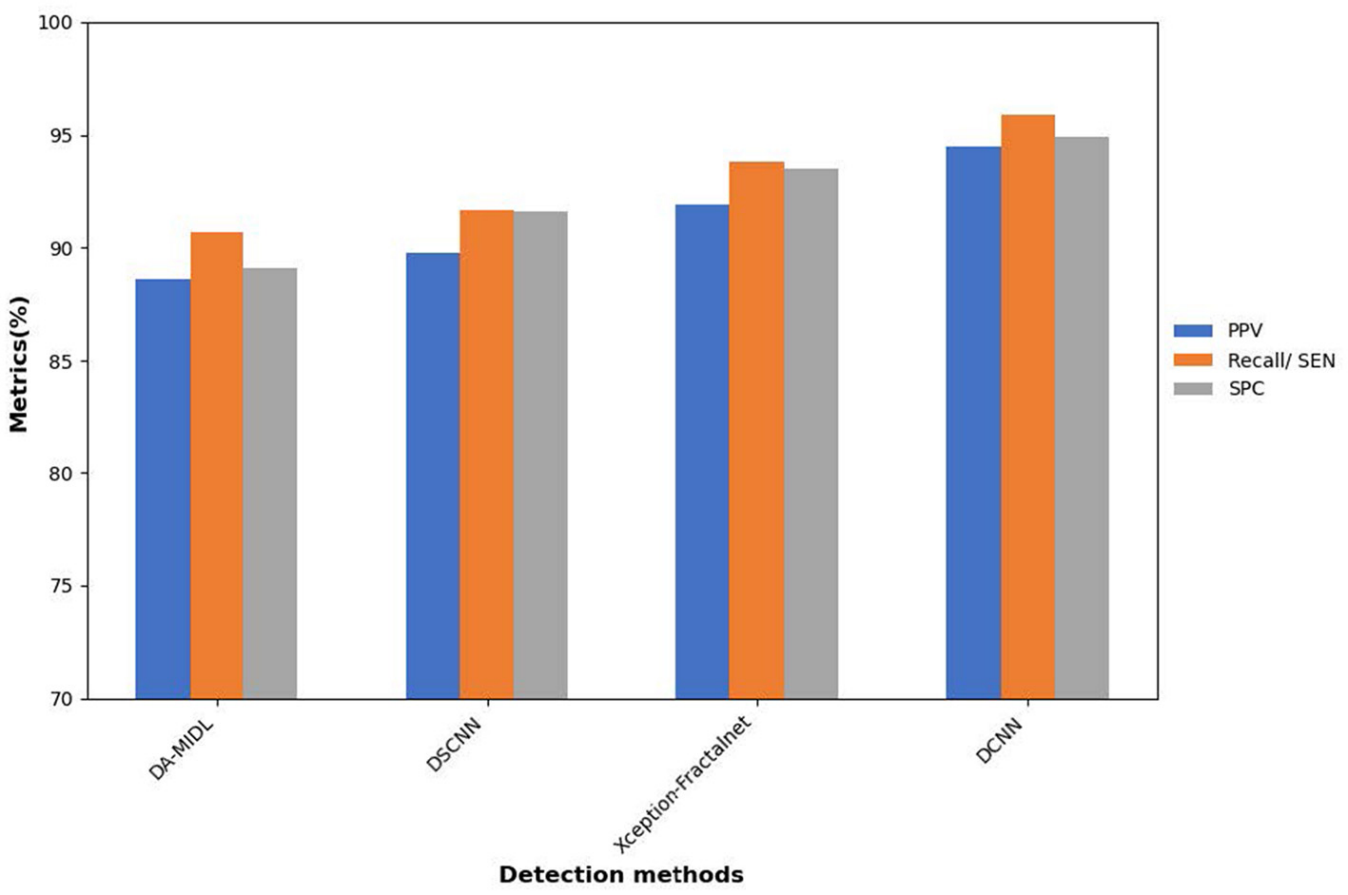
Results comparison of detection approaches.

Figure 13 shows a comparison of detection approaches based on PPV, SEN, and SPC. The proposed classifier has the highest PPV, SEN, and SPC of 94.55, 95.86, and 94.93%. DA-MIDL, DSCNN, and Xception-Fractalnet achieve the lowest PPV of 88.61, 89.78, and 91.89%, respectively. DA-MIDL, DSCNN, and Xception-Fractalnet achieve the lowest SEN of 90.67, 91.65, and 93.73%, respectively. DA-MIDL, DSCNN, and Xception-Fractalnet achieve the lowest SPCs of 89.08, 91.57, and 93.45%, respectively (see Table 4).

**Table 4:** Evaluation results comparison of AD detection methods.

Methods/results (%)	PPV	Recall/SEN	SPC	F1-score	ACC	AUC
DA-MIDL	88.61	90.67	89.08	89.63	90.51	91.74
DSCNN	89.78	91.65	91.57	90.71	92.75	93.28
Xception-Fractalnet	91.89	93.73	93.45	92.80	94.08	94.61
DCNN	94.55	95.86	94.93	95.21	95.87	96.45

**Figure 14:**
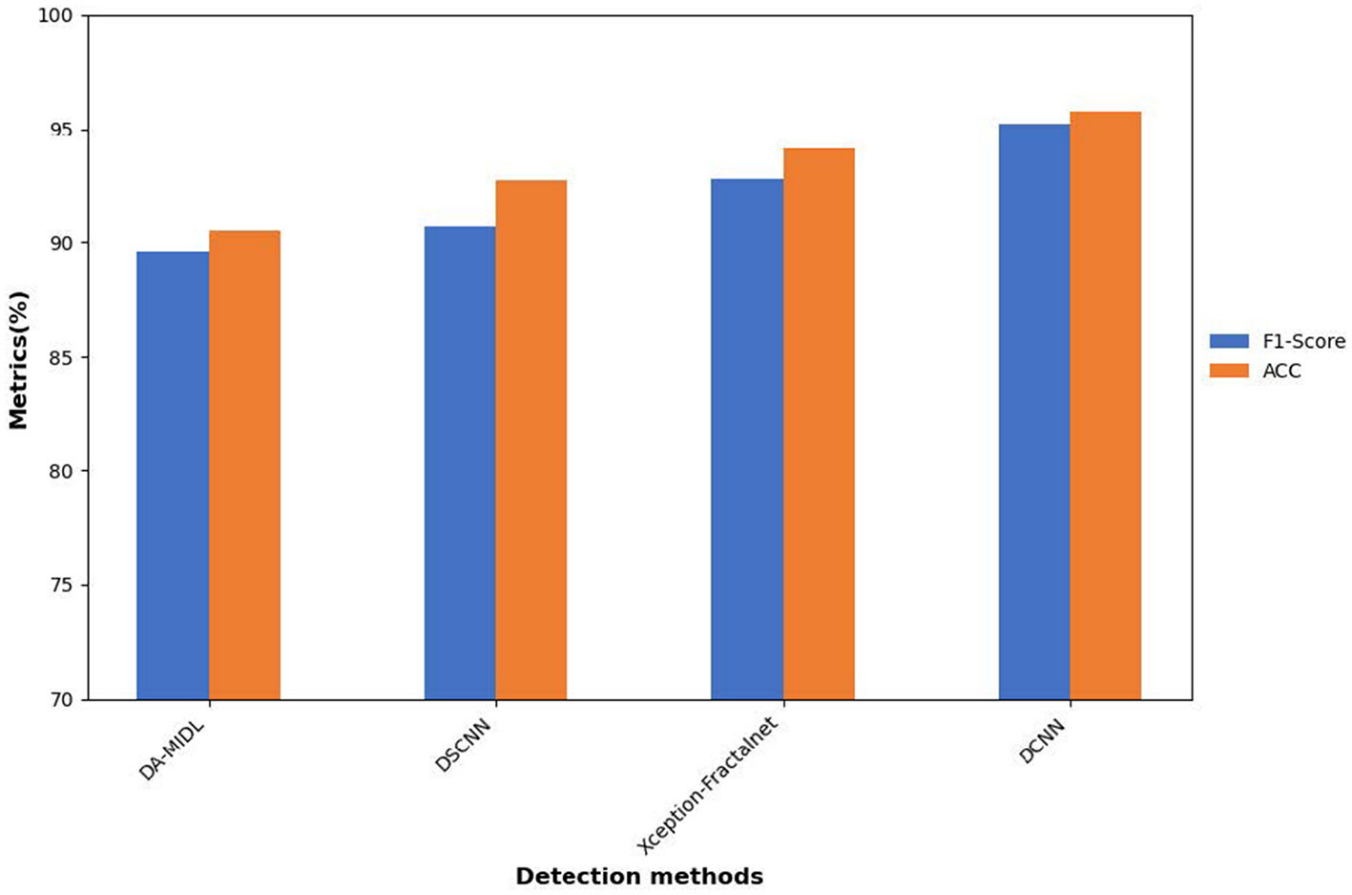
F1-Score and ACC comparison of detection methods.

DA-MIDL, DSCNN, Xception-Fractalnet, and DCNN with respect to f1-score and ACC are illustrated in Figure 14. The proposed classifier achieves the highest F1-score and ACC of 95.21 and 95.87%, respectively. DA-MIDL, DSCNN, and Xception-Fractalnet achieve the lowest f1-scores of 89.63, 90.71, and 92.80%, respectively. DA-MIDL, DSCNN, and Xception-Fractalnet achieve the lowest ACCs of 90.51, 92.75, and 94.08%, respectively (see Table 4).

**Figure 15:**
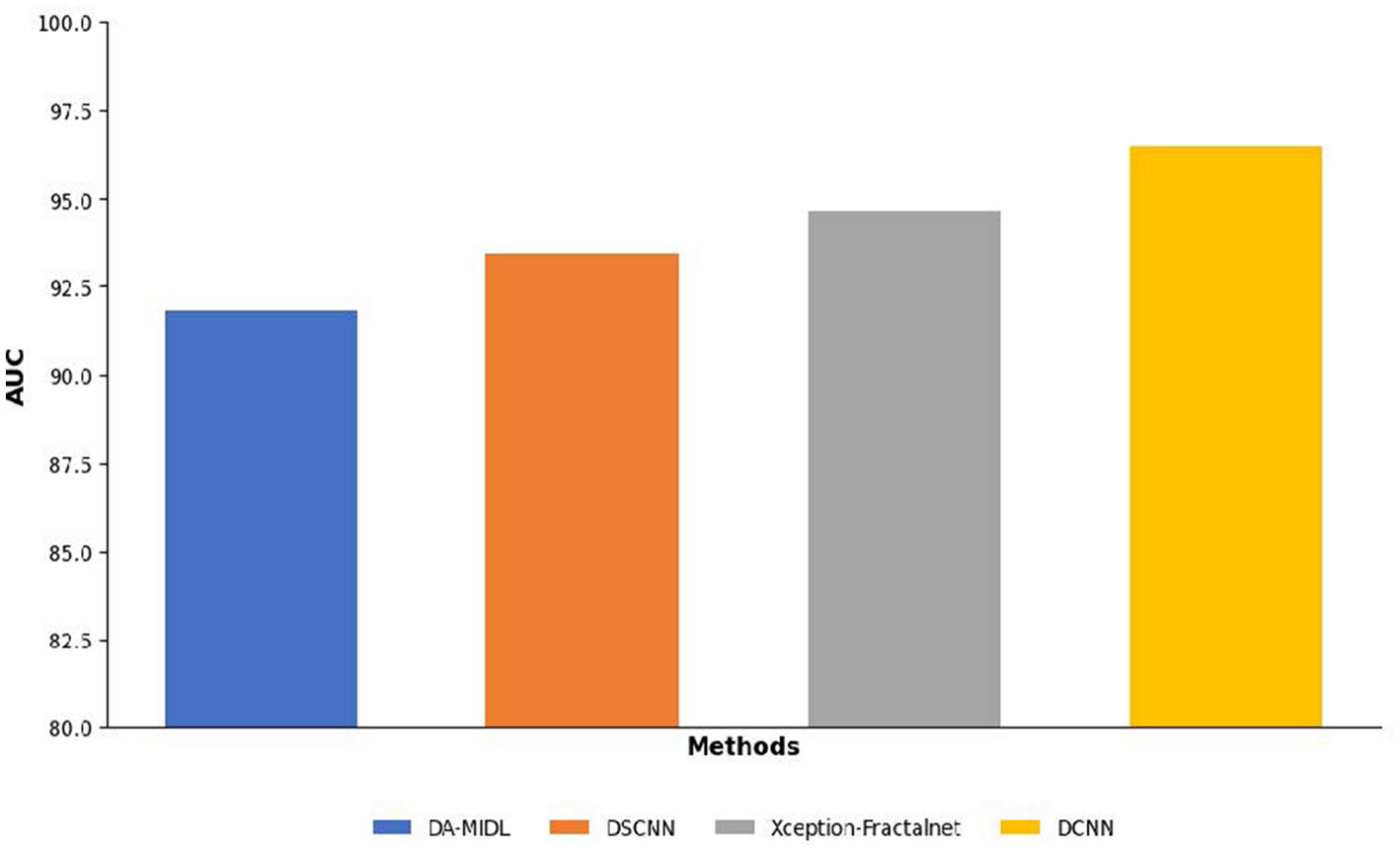
AUC comparison of detection methods.

DA-MIDL, DSCNN, Xception-Fractalnet, and DCNN with respect to AUC are illustrated in Figure 15. The proposed classifier achieves the highest AUC of 96.45%; DA-MIDL, DSCNN, and Xception-Fractalnet achieve the lowest AUCs of 91.74, 93.28, and 94.61%, respectively. DA-MIDL, DSCNN, and Xception-Fractalnetachieve the lowest AUCs of 4.71% 3.07, and 1.84%, respectively, compared to the proposed method (see Table 4).

## 5 Conclusion and future work

In this study, a DCNN is introduced that uses an MRI dataset to classify samples as AD/non-AD. Pre-processing using an adaptive mean filter, image denoising of the MRI input dataset, Ensemble Deep Learning Denoising (EDLD), feature extraction by a CNN, and classification are performed using a DCNN, and performance is evaluated. Ensemble Deep Learning Denoising (EDLD) is a combination of three deep learning methods. Introducing the attention-guided convolution neural network (AGCNN) for image denoising: To enhance alignment and reduce noise, AGCNN additionally learns from areas particular to AD disease and uses a global branch to make substitutions for the local branch's missing discriminatory signals. In an Adaptive Denoising Autoencoder (ADAE), the encoder learns pixel representations, and the decoder performs image reconstruction. The Gaussian Deep Belief Network (GDBN) is structured into multiple layers, each containing a varying number of neurons, and is built from Gaussian Restricted Boltzmann Machines (GRBMs). Once the individual has denoised the training image dataset to train the models, they are evaluated on a separate testing dataset.

An average of each model's weighted PSNR is computed to provide the final denoised image. Image augmentation serves not only to correct the unequal distribution of data through scaling, affine transformations, translation, gamma correction, and rotation, but also to improve the robustness of machine learning models. To distinguish between the foreground and the background, the OTSU thresholding technique is refined using the Levy Grasshopper Optimization Algorithm (LGOA), which sets the threshold to the minimum variance of the two resulting clusters. The VGG16 model is pre-trained to recognize pictures, and CNNs automatically encode raw images. SEN, SPC, PPV, F1, ACC, and AUC measure model effectiveness. These models need clinical evaluation to fine-tune hyperparameters to be most effective. Given the volume of data involved in diagnosing brain disease, the diverse sources of data, and the constant influx of sensor data, data processing systems face significant challenges in processing and storing the resulting data, which remains unexplored.

## Data Availability

Publicly available datasets were analyzed in this study. This data can be found at: OASIS MRI dataset (https://sites.wustl.edu/oasisbrains/).
